# Apolipoproteins L1-6 share key cation channel-regulating residues but have different membrane insertion and ion conductance properties

**DOI:** 10.1016/j.jbc.2021.100951

**Published:** 2021-07-10

**Authors:** Jyoti Pant, Joseph A. Giovinazzo, Lilit S. Tuka, Darwin Peña, Jayne Raper, Russell Thomson

**Affiliations:** 1 Department of Biological Sciences, Hunter College, City University of New York, New York, New York, USA; 2 Department of Biochemistry and Molecular Genetics, School of Medicine, University of Colorado Anschutz Medical Campus, Aurora, Colorado, USA; 3 PhD Program in Biochemistry, The Graduate Center of the City University of New York, New York, New York, USA

**Keywords:** apolipoprotein L, *APOL* gene family, APOL protein family, ion channels, innate immunity, kidney disease, cell death, pH-gating, voltage-dependent ion channel, APOL, apolipoprotein L, HDL, high-density lipoprotein, HIV, human immunodeficiency virus, IRG, interferon regulated gene, LDH, lactate dehydrogenase, MID, membrane-insertion domain, PLR, pore-lining region, TLF, trypanosome lytic factor

## Abstract

The human apolipoprotein L gene family encodes the apolipoprotein L1–6 (APOL1–6) proteins, which are effectors of the innate immune response to viruses, bacteria and protozoan parasites. Due to a high degree of similarity between APOL proteins, it is often assumed that they have similar functions to APOL1, which forms cation channels in planar lipid bilayers and membranes resulting in cytolytic activity. However, the channel properties of the remaining APOL proteins have not been reported. Here, we used transient overexpression and a planar lipid bilayer system to study the function of APOL proteins. By measuring lactate dehydrogenase release, we found that APOL1, APOL3, and APOL6 were cytolytic, whereas APOL2, APOL4, and APOL5 were not. Cells expressing APOL1 or APOL3, but not APOL6, developed a distinctive swollen morphology. In planar lipid bilayers, recombinant APOL1 and APOL2 required an acidic environment for the insertion of each protein into the membrane bilayer to form an ion conductance channel. In contrast, recombinant APOL3, APOL4, and APOL5 readily inserted into bilayers to form ion conductance at neutral pH, but required a positive voltage on the side of insertion. Despite these differences in membrane insertion properties, the ion conductances formed by APOL1-4 were similarly pH-dependent and cation-selective, consistent with conservation of the pore-lining region in each protein. Thus, despite structural conservation, the APOL proteins are functionally different. We propose that these proteins interact with different membranes and under different voltage and pH conditions within a cell to effect innate immunity to different microbial pathogens.

The apolipoprotein L (*APOL*) gene family was identified in 2001 (1). The first APOL protein identified was APOL1, which led to the identification of five other APOLs, APOL2-6 (1, 2, 3). *APOL1-4* forms a gene cluster that is separated from *APOL5* and *6* within 619 kb on chromosome 22 (Fig. 1*A*). Collectively, these genes make up the *APOL* gene family (1, 2, 4). The APOL1-4 gene cluster is present only in humans and higher primates (4, 5). Based on previous studies, *APOL1-4* arose during recent primate evolution from a single ancestral gene due to gene duplication, while *APOL5* and *APOL6* are more ancient in origin (4, 5).

**Figure 1:**
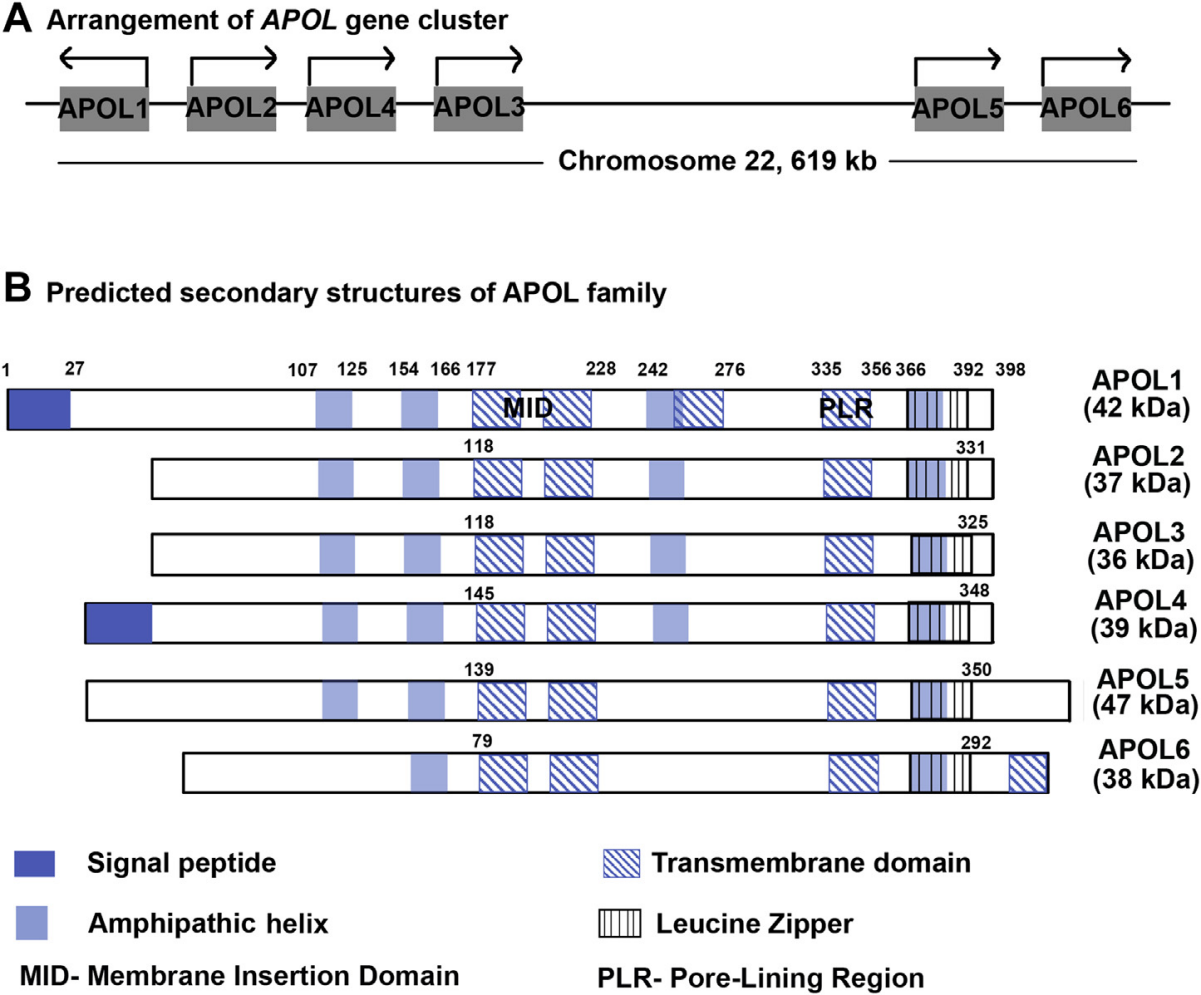
***APOL* gene family and their predicted domain structures.***A*, *APOL* gene family on chromosome 22. *B*, predicted domain structure of APOL protein family along with expected molecular weight.

The *APOL* genes are hypothesized to be innate immunity genes because they are rapidly evolving in primates, and their expression is upregulated by proinflammatory signals (4, 6). In fact, individual overexpression of any one of *APOL1, 2*, and *6* genes was shown to inhibit the replication of various viruses (6, 7). Of the six, *APOL1* is the most studied and well characterized. APOL1 is the only protein that is secreted from cells due to the presence of an N-terminal signal peptide (3, 8), and it circulates in serum on a subfraction of high-density lipoproteins (HDL). The HDL complexes containing APOL1 make up 1% of the total HDL pool in the plasma and have been investigated for their antiparasitic activity against kinetoplastid parasites *Trypanosoma brucei* and *Leishmania* sp (8, 9, 10). Two recently evolved human APOL1 variants (renal-risk variants) are associated with increased risk of chronic kidney diseases (11, 12). Therefore, in recent years, APOL1 has been extensively studied in terms of its structure, function, and cellular localization.

APOL1 is a cation channel forming protein that inserts into lipid bilayers at acidic pH and forms a closed-state ion channel. These channels are gated by pH, such that they open upon pH neutralization and allow the flux of cations across the bilayer (13). Therefore, according to the proposed model of trypanosome lysis, HDL is first endocytosed into endosomes where APOL1 inserts into the endosomal membrane at acidic pH to form a closed ion channel. During membrane recycling, the membrane-inserted APOL1 ion channel reaches the plasma membrane where it is exposed to neutral pH (extracellular milieu), allowing for APOL1 ion channel opening. This in turn causes an ionic imbalance, followed by osmotic swelling and lysis of the parasite (13, 14, 15). This cytolytic effect, along with the swelling, is also observed in mammalian cells transiently transfected with APOL1, suggesting that APOL1 may function similarly in trypanosomes and human cells (16, 17, 18, 19). However, in stable transfected cells, which produce less protein than the transient system, only the renal-risk variants, namely G1 (S342G, I384M; rs73885319 and rs60910145) and G2 (del388:389NY, rs71785313) are cytotoxic, whereas the most common APOL1 variant is not (19, 20). To account for these data, a dose-dependent gain-of-function model of cytotoxicity has been proposed. According to this model, APOL1 is capable of insertion into the membranes of acidic Golgi and/or secretory vesicles during transit through the secretory pathway, allowing for the opening of APOL1 cation channels in the plasma membrane of human cells. Increases in expression (*e.g.*, overexpression or upregulation by interferon) and increased channel-forming propensity (in the case of renal-risk variants) allows for increased channel formation and therefore greater cation influx and cytotoxicity (19, 21).

Little is known about the ion-channel properties of the other APOL family proteins, although recombinant APOL3 was recently reported to produce an ion flux in artificial lipid bilayers (22). In addition, APOL6 caused apoptosis when overexpressed in a human cancer cell line (23). Due to similarities in the amino acid sequences, we and others hypothesize significant functional conservation between members of the APOL protein family. Some studies have shown differential expression of APOLs upon infection with different microbes such as viruses, *Mycobacterium* and *Listeria* (6, 7, 24, 25). In addition, *APOL* genes show signatures of positive selection, providing additional evidence for their role in innate immunity (4). However, until now, there has been no reported attempt to characterize and compare the basic cellular and biochemical functions of the APOL proteins. In order to understand the function of these proteins, we compared the cytotoxic effects of APOL proteins in human cells and their ability to generate an ion conductance in planar lipid bilayers.

## Results

Of the six human *APOL* genes (Fig. 1*A*), *APOL1* is the most studied; the APOL1 protein is known for its ability to kill some *Trypanosomatids* and for its association with chronic kidney disease in humans (8, 26). Here, we investigated the function of other human APOL proteins using APOL1 as a template.

Using a comparative analysis of human, baboon, and synthetic APOL1 mutants, Schaub *et al.* (27) showed evidence that APOL1 contains four transmembrane domains in the open cation channel conformation (Fig. 1*B*, blue hatched boxes). The first two transmembrane domains are important for pH-dependent insertion into target membranes (membrane-insertion domain; MID), whereas the fourth transmembrane domain (pore-lining region; PLR) is important for pH-dependent ion-channel gating and cation *versus* anion selectivity of the channel (27). We used transmembrane prediction servers and comparison with APOL1 to annotate the domain structures of APOL1-6 (2, 27). We found a high degree of structural similarity among all APOL proteins, with APOL1 through APOL4 more similar than APOL5 and APOL6 (Fig. 1*B*). Notably, APOL1 has an extra putative transmembrane domain (257–273) that was not predicted in the case of any other APOL protein (Figs. 1*B* and S1). Likewise, APOL6 has a unique predicted transmembrane structure at the C-terminal end of the protein (Figs. 1*B* and S1). Only APOL1 and APOL4 have isoforms with a predicted signal peptide sequence (Fig. S2). However, conservation of the MID and PLR transmembrane domains in all APOL proteins suggests functional similarity.

### APOL1, APOL3, and APOL6 are cytotoxic when overexpressed in cells

**Figure 2:**
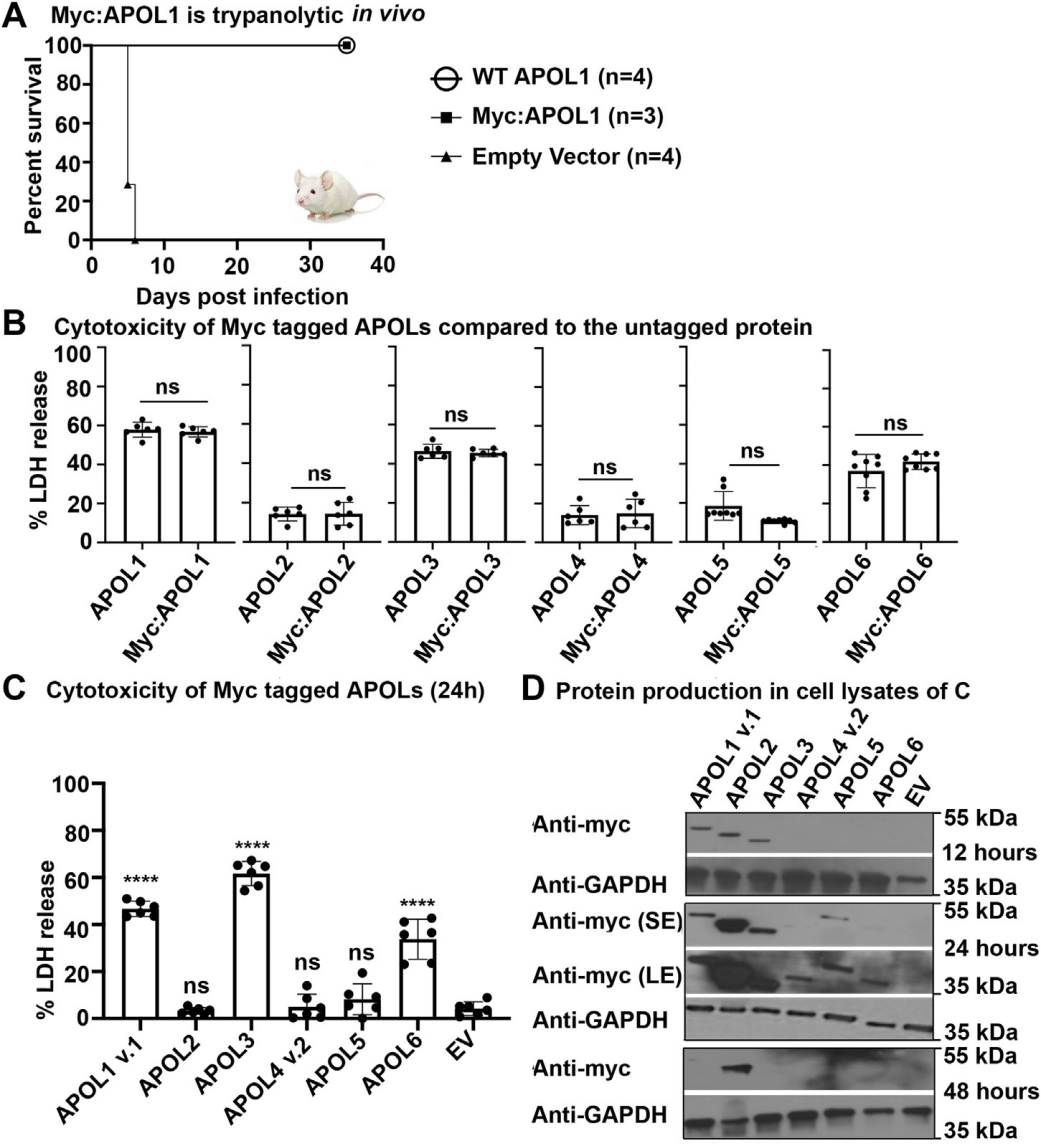
**APOL1, APOL3, and APOL6 are cytotoxic when overexpressed in cells.***A*, transient transgenic mice were created by injecting 25 μg of plasmid DNA containing *APOL1*, *Myc*-tagged *APOL1*, or no insert (empty vector) in a single plasmid pRG977 by hydrodynamic gene delivery (HGD) followed by infection by 5000 *T.b. brucei* (2 days post HGD). Mice were monitored for parasitemia and survival for 35 days post infection. The data represents one typical experiment that has been repeated two times ∗∗*p* = 0.0004 (Log-rank test). *B*, HEK293 cells were transfected with Lipofectamine-3000. Cytotoxicity was measured by assaying LDH release (24 h post transfection) from HEK293 cells transfected using 100 ng of *APOL1–APOL6* (wild-type) or Myc-tagged *APOL1–APOL6* in plasmid vector pRG977. The percentage of LDH released was normalized to that released from nontransfected cells treated with transfection reagents. ns: not significant, Student's *t* test was performed. Results from a single representative experiment from three independent experiments are shown as Mean ± SD. *C*, Myc-tagged *APOL*s in pRG977 were transfected into HEK293 cells and release of LDH was measured in the media 24 h post transfection (peak time of protein expression) ∗∗∗∗*p* < 0.0001 and ns: not significant, Shapiro–Wilk Normality test for normality distribution and ANOVA, multiple comparison with Bonferroni correction was performed. Results from a single representative experiment from three independent experiments are shown as Mean ± SD. *D*, APOL protein levels in the cell lysates were assessed by SDS-PAGE followed by immunoblotting for anti-Myc antibody at 12, 24, and 48 h post-transfection. LE, long exposure; SE, short exposure.

In order to investigate the potential cytotoxic functions of APOL1-6, we first generated expression constructs to provide an Myc-tag at the N-terminus of each protein (Fig. S2), or in the case of APOL1 and APOL4, just C-terminal of the reported signal peptide cleavage site (3). In the transient transfection mouse model transfected by hydrodynamic gene delivery, the N-terminally tagged APOL1 protected against African trypanosome *T. brucei brucei* infection similar to wild-type APOL1 (Fig. 2*A*). Importantly, the N-terminally tagged APOLs functioned similarly to untagged APOL proteins when produced in human HEK293 cells and assayed for cytotoxicity (Fig. 2*B*). In the overexpressing cells, we observed significant release of lactate dehydrogenase (LDH) by cells expressing *APOL1*, *APOL3*, and *APOL6* at 24 h posttransfection when there is peak production of the proteins (Fig. 2, *B–D*). Although we saw marked differences in the cellular abundance of different APOL proteins (Fig. 2*D*), these differences could not fully account for the divergent levels of cytotoxicity observed (Fig. 2*C*). For example, the amount of APOL2 protein was higher than all other APOLs, yet no cytotoxicity was observed, and the APOL6 level was as low as APOL4, yet significant LDH release was measured in the case of APOL6, but not APOL4 (Fig. 2, *C* and *D*). Under a light microscope, the cells producing APOL1 and APOL3 (Myc:APOL1 and Myc:APOL3) appeared swollen at 24 h (Fig. S3), whereas cells producing APOL6 or Myc:APOL6 did not visibly swell (Fig. S3). Consistent with the LDH assay results, there were fewer cells visible when *APOL1*, *APOL3*, and *APOL6* were being expressed compared with *APOL2*, *APOL4*, and *APOL5* (Figs. 2, *B* and *C* and S3). Thus, our results suggest that *APOL1*, *APOL3*, and *APOL6* are cytotoxic when overexpressed in HEK293 cells but *APOL2*, *APOL4*, and *APOL5* are not.

**Figure 3:**
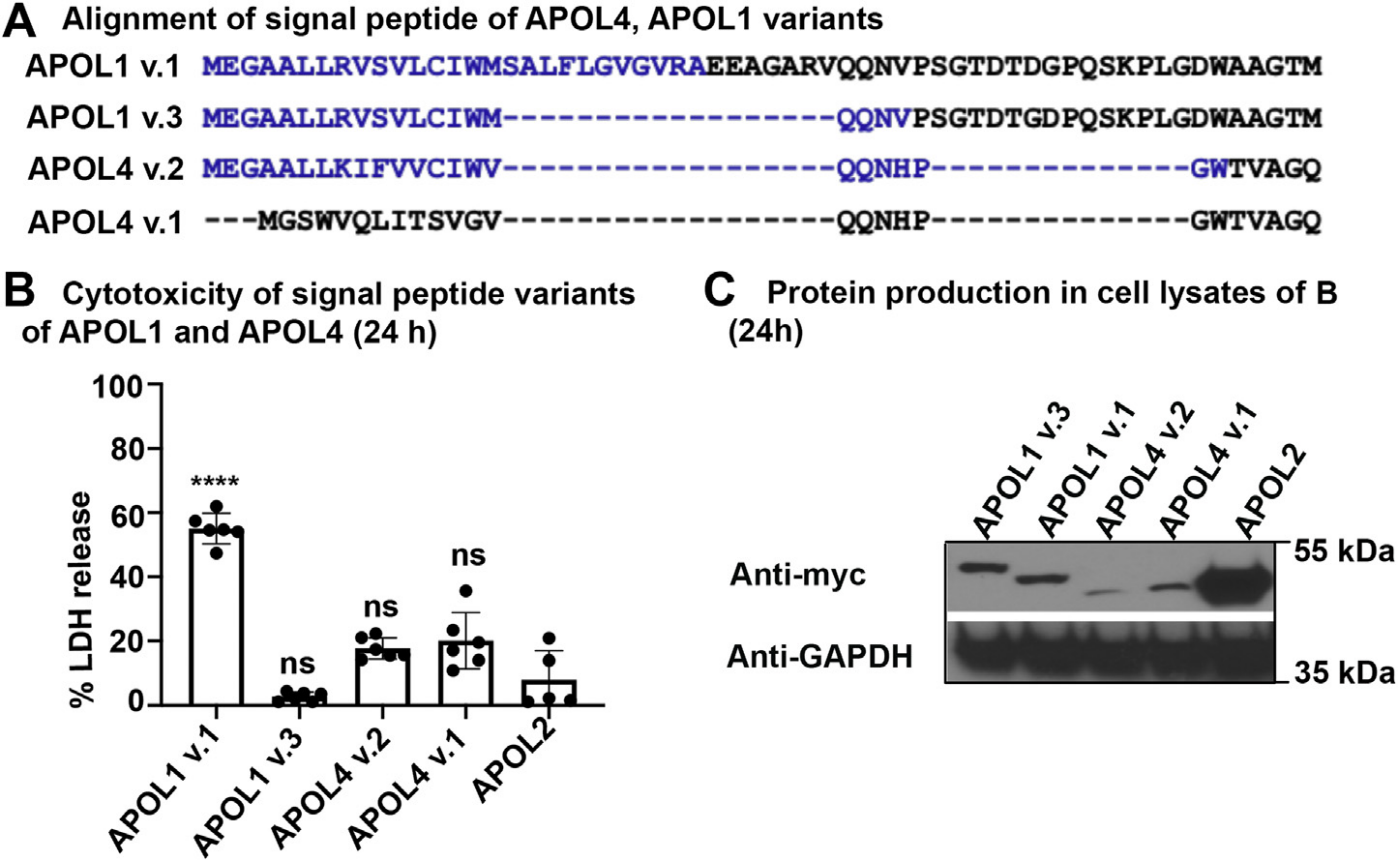
**Cytotoxicity of APOL1 is dependent on predicted signal peptide.***A*, alignment of APOL1 v.1, APOL1 v.3, APOL4 v.1, and APOL4 v.2 N-terminal regions. *Blue text* indicates signal peptides as predicted by the SignalP-5.0 server (28). *B*, HEK293 cells were transiently transfected with APOL1 v.1, APOL1 v.3, APOL4 v.2, APOL4 v.1, and APOL2. Cytotoxicity was measured by assaying LDH released into the media 24 h post transfection. ∗∗∗∗*p* < 0.0001. Shapiro–Wilk Normality test for normality distribution and ANOVA, multiple comparison with Bonferroni correction was performed. Results from a single representative experiment from three independent experiments are shown as Mean ± SD. *C*, lysates of the same cells represented in *panel D* were separated by SDS PAGE and probed by immunoblotting with anti-Myc antibody at 24 h posttransfection.

In addition to the most abundant APOL1 isoform (variant v.A also called v.1) used above, there exists a minor splice variant (v.C also called v.3) that lacks a strong signal peptide prediction using SignalP-5.0 software (Figs. 3*A* and S2*B*) (28, 29). To test the effect of the APOL1 signal peptide, we expressed the two isoforms in HEK293 cells. LDH release from cells producing APOL1 v.3 was reduced compared with APOL1 v.1 and was similar to those cells producing APOL2 that is noncytotoxic to the transfected cells (Fig. 3*B*). This occurred despite similar protein levels between the two APOL1 isoforms (Fig. 3*C*), suggesting that the signal peptide is required for significant cytotoxicity. APOL4 is reported to have two isoforms—isoform 2 (v.2), which has a weak predicted signal peptide; and isoform 1 (v.1), which lacks any hint of an N-terminal signal peptide (Figs. 3*A* and S2*B*). Therefore, we tested the cytotoxicity of these naturally occurring APOL4 isotypes. Cells producing either of these two APOL4 isoforms showed very low LDH release (Fig. 3, *B* and *C*) and hence no measurable cytotoxicity. We conclude that APOL1, but not APOL4 cytotoxicity, was affected by the presence of a signal peptide when overexpressed in HEK293 cells.

**Figure 4:**
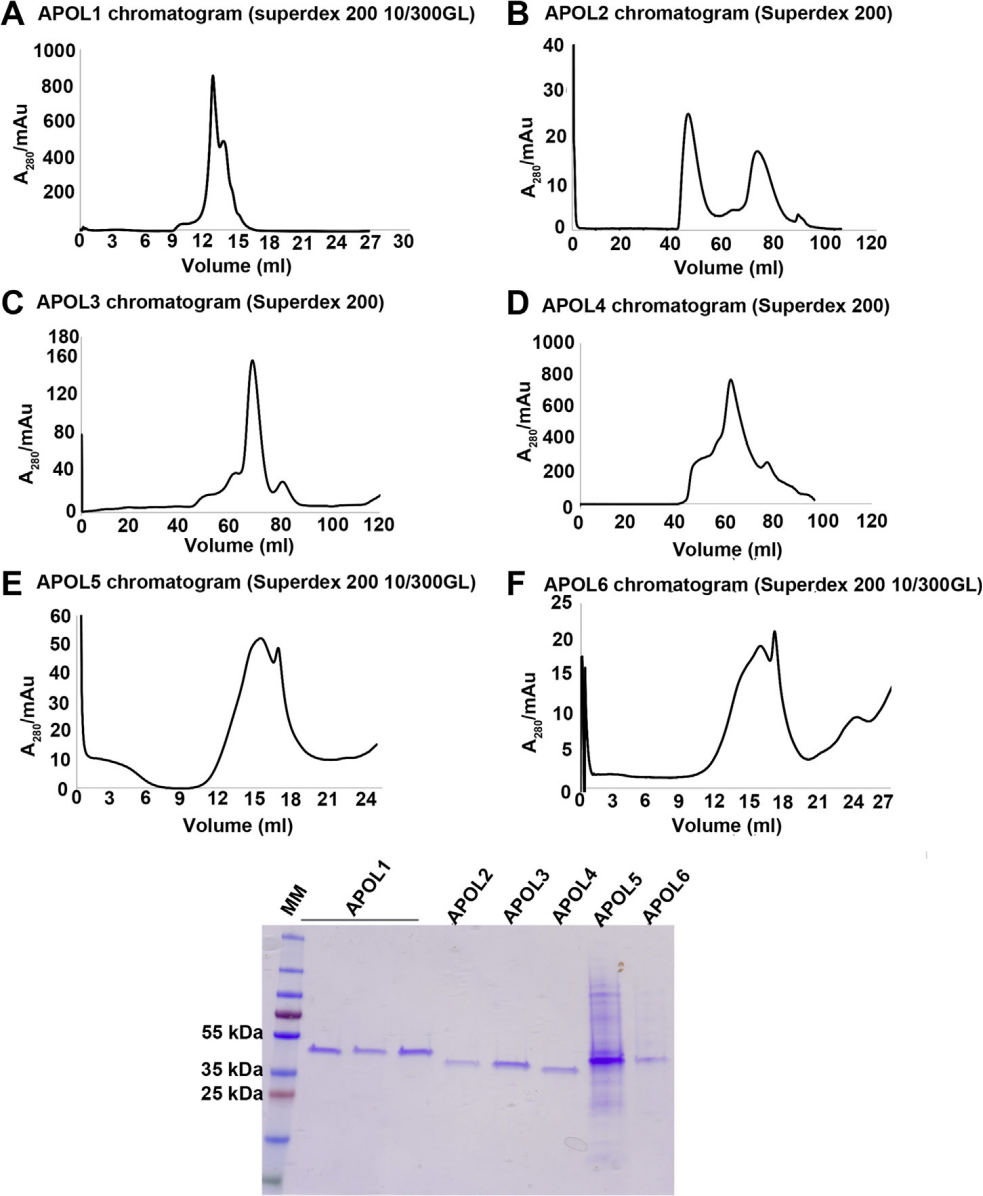
**Purification of recombinant APOL1–6 proteins from bacteria.** His-tagged APOL1–6 were produced in *E. coli* as inclusion bodies that were solubilized in zwittergent 3-14, and affinity purified using nickel columns as described in detail in Experimental procedures. The enriched proteins were further purified by size-exclusion chromatography. *Panels A–F* are chromatograms of the size-exclusion column eluates showing fractions in ml (*x*-axis) and absorbance at 280 (*y*-axis) of (*A*) APOL1, (*B*) APOL2, (*C*) APOL3, (*D*) APOL4, (*E*) APOL5, (*F*) APOL6. Below is a Coomassie stained SDS-PAGE gel of the purified recombinant APOL proteins.

Taken together, our results show that APOL1, APOL3, and APOL6 are cytotoxic when overexpressed in HEK293 cells. Furthermore, cells producing APOL1 and APOL3 were morphologically distinct from cells producing APOL6. To determine if these differences in cytotoxicity between APOLs could be related to differences in ion-channel forming properties, we tested recombinant APOL proteins (Fig. 4) for their ability to generate an ion conductance in artificial planar lipid bilayers.

### APOL1 and APOL2 insert into lipid bilayers at acidic pH and form pH-gated ion channels

**Figure 5:**
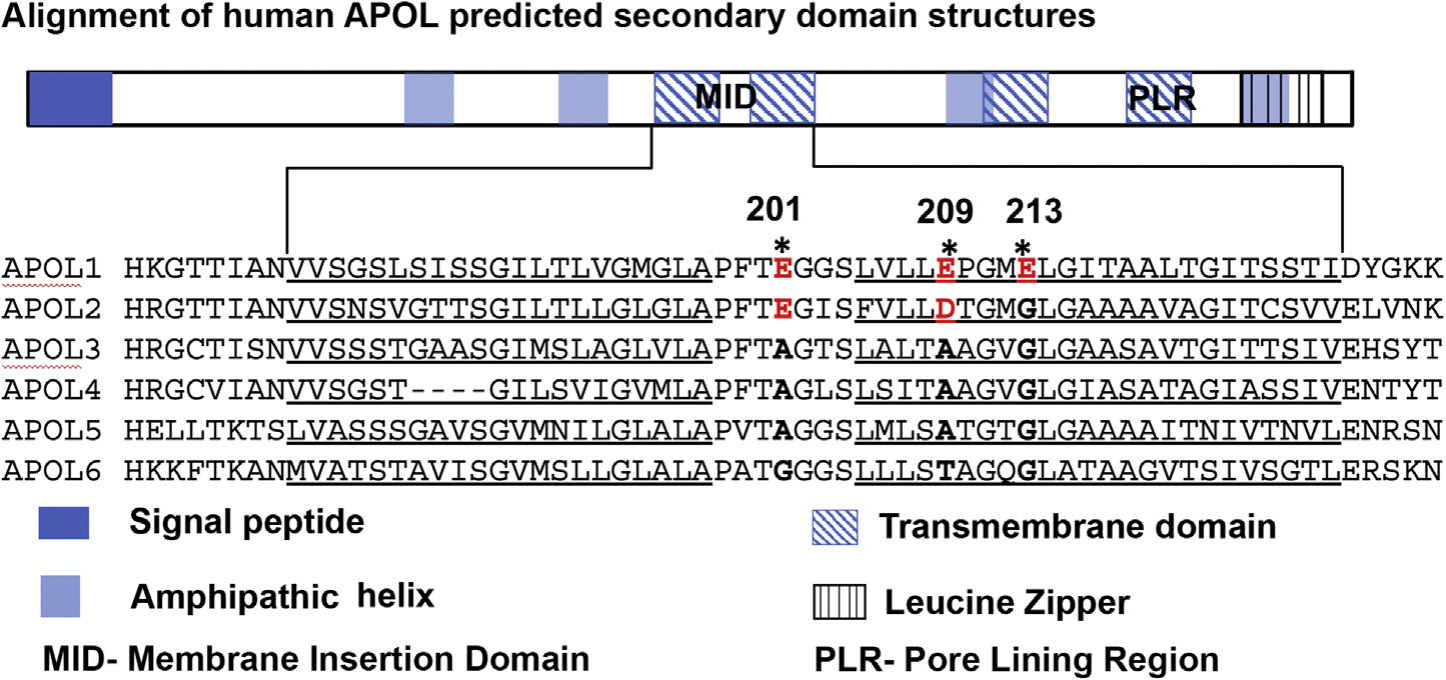
**Alignment of human APOL membrane-insertion domains (MID).** Above, schematic of human APOL1 domain structure model according to Schaub *et al.* (27). Below, alignment of APOL1-6 MIDs, which encompasses the first and second transmembrane domains, shows that the negatively charged amino acid residues at position 201 (E, glutamic acid) and 209 (E/D, glutamic acid/aspartic acid) of APOL1 are conserved in APOL2 but not in APOL3-6. ∗represents the negatively charged residues in the membrane-insertion domain (MID) in APOL1 at positions 201, 209, and 213.

**Figure 6:**
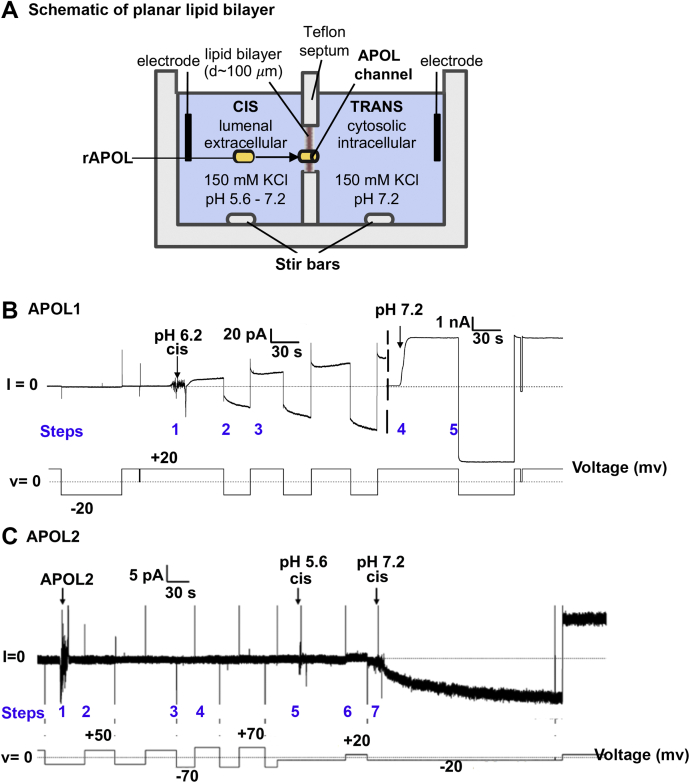
**APOL1 and APOL2 insert into planar lipid bilayers at acidic pH to form a pH-dependent ion conductance.***A*, schematic of the planar lipid bilayer system. *B* and *C*, planar lipid bilayers were formed between symmetric solutions of bilayer buffer, pH 7.2 (see Experimental procedures). The current (upper trace) was recorded, as the voltage was set by the experimenter (lower trace). *B*, prior to the start of the record, 1 μg (23.8 nmol) APOL1 was added to the *cis* side of the bilayer and the *cis* pH was adjusted to pH 6.2 (step 1), which resulted in a relatively minor increase in bilayer current magnitude, *i.e.*, ion flow (note 20 pA scale). Changing voltage to +20 mV or –20 mV (steps 2 and 3) had little effect on current magnitude. The *cis* side was then adjusted to pH 7.2 (step 4) as indicated, which resulted in a several hundred-fold increase in the current magnitude, due to the opening of pH-gated channels (note 1 nA scale). Changing voltage to –20 mV (step 5) had little effect on current magnitude. C. 500 ng (13.47 nmol) APOL2 was added on the *cis* side of the bilayer at pH 7.2 (step 1). No change in current was observed as the voltage was changed between +50 mV, –70 mV, and +70 mV (steps 2, 3, and 4). The *cis* side was then adjusted to pH 5.6 (step 5), which resulted in a small increase in bilayer current magnitude. Voltage was adjusted to +20 mV (step 6). After the *cis* side was then returned to pH 7.2 with KOH (step 7), there was a further 15-fold increase in current magnitude, indicative of pH gating.

APOL1, the most studied of all the APOLs, is known to insert into planar lipid bilayers and form a closed ion channel, when the pH on the *cis* side (*i.e.*, the side the APOL is added to the chamber) is acidic (mimicking the trypanosome endocytic compartment or the secretory pathway of human cells including podocytes) and the pH on the *trans* side (the side opposite to where APOL is added) is neutral (mimicking the cytoplasmic compartment). This closed channel subsequently opens to allow the conductance of cations when the *cis* pH is neutralized (mimicking the extracellular side of the plasma membrane), while keeping the *trans* (cytoplasmic face) pH neutral (13). We propose that other APOL proteins would form ion channels, because their domain structures are largely conserved, including the transmembrane domains (Fig. 1*B*). To investigate this, we compared the amino acids of the conserved first and second transmembrane regions, which together form the membrane-insertion domain (MID) (Fig. 5) (27). As previously reported, negatively charged glutamic acid residues in the APOL1 MID play an important role in pH-dependent channel formation by APOL1 in planar lipid bilayers, suggesting that they must be protonated/neutralized at acidic pH to allow membrane insertion and channel formation to occur (13). The amino acid alignment shows that other than APOL1, only APOL2 shares the negatively charged amino acid residues corresponding to APOL1 E201 and E209, whereas the other APOLs completely lack charged amino acid residues in the MID (Fig. 5). Utilizing the planar lipid bilayer setup, with alteration of the *cis* pH (Fig. 6*A*), we found that APOL2, like APOL1, inserted into lipid bilayers at acidic pH 5.6 (step 5), but not at neutral *cis* pH, as detected by a relatively small change in bilayer ion conductance at pH 5.6 (Fig. 6, *B* and *C*). This APOL2-induced ion conductance increased by 15-fold after subsequent neutralization of *cis* compartment with KOH compared with a 100-fold increase with APOL1 (Fig. 6, *B* and *C*) (13). Thus, like APOL1, APOL2 appears to insert into lipid bilayers at acidic pH to form a closed ion channel that opens on subsequent neutralization of the pH. However, it should be noted here that unlike APOL1, which localizes to the ER lumen and secretory pathway due to an N-terminal signal peptide, we do not know where in the cytosol (neutral pH) APOL2 would be exposed to acidic pH in the cell, because APOL2 lacks a signal peptide and was previously shown to localize to the cytoplasmic face of the ER membrane (30).

### APOL3, APOL4, and APOL5 increase the ion permeability of planar lipid bilayers at neutral pH and in a voltage-dependent manner

**Figure 7:**
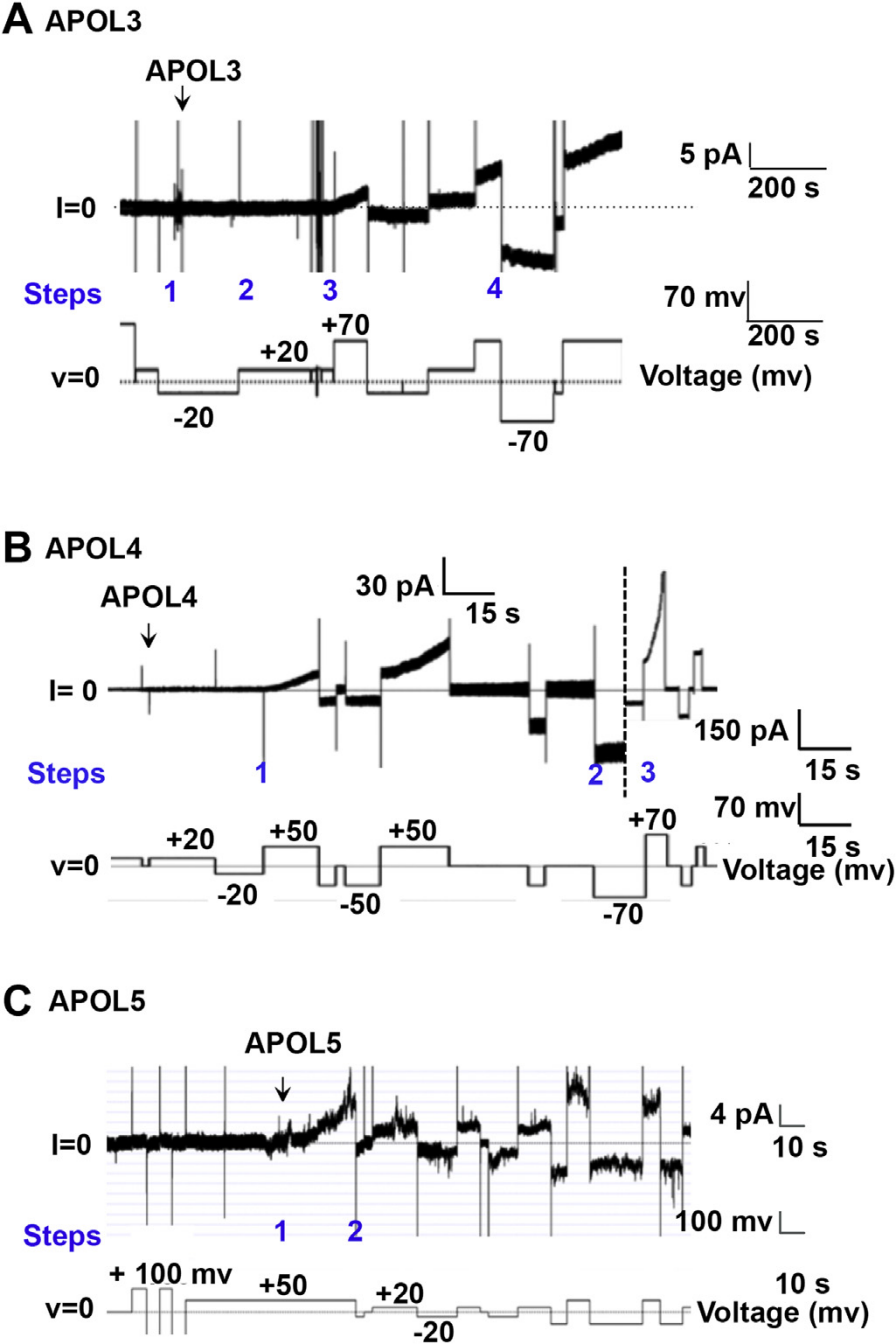
**APOL3, APOL4, and APOL5 form a voltage-dependent ion conductance in planar lipid bilayers at neutral pH.** Planar lipid bilayers were formed between symmetric solutions of bilayer buffer, pH 7.2. In each experiment, the current was recorded (upper trace) as the voltage was set by the experimenter (lower trace). *A*, with the voltage held at –20 mV, APOL3 (600 ng, 13.55 nM) was added to the *cis* side (step 1). No change in current was observed, either at –20 mV, or when the voltage was switched to +20 mV (step 2). However, after adjustment to +70 mV (step 3) there was a linear increase in current, likely due to the gradual incorporation of protein into the bilayer. This increase in current magnitude was sustained after switching to –70 mV (step 4, note 5 pA scale). *B*, before the start of the record, APOL4 (600 ng, 15.3 nM) was added to the *cis* side. No change in current was observed until after the voltage was increased to +50 mV (step 1), when there was a gradual increase in current, likely due to incorporation of protein into the bilayer. No further change in current was observed at –70 mV (step 2), but the rate of current change was further increased at +70 mV (step 3, note 150 pA scale). C. With the voltage held at +50 mV APOL5 (840 ng, 17.85 nM) was added to *cis* side (step 1), resulting in a rapid increase in current. After switching the voltage to –20 mV (step 2), there was little further increase in ion conductance (note 4 pA scale).

Next, we characterized the formation of an ion conductance by APOL3–6, which all have the small uncharged nonpolar amino acids alanine (A) or glycine (G) substituted at the positions corresponding to negatively charged glutamic acid residues E201, E209, and E213 in the APOL1 MID (Fig. 5). We predicted that these changes, negatively charged to neutral amino acids, would permit channel formation at neutral pH. However, addition of APOL3 and APOL4 to the bilayer at neutral pH 7.2 did not result in ion conductance until the voltage across the bilayer was adjusted to a positive value (Fig. 7, *A* and *B*). In the case of APOL3 (Fig. 7*A*), we observed a continuous and linear increase in ion conductance after the voltage was increased from +20 to +70 mV (step 3), likely due to voltage-dependent incorporation of protein into the membrane from the *cis* solution (the side where APOL proteins were added). However, this gradual increase in bilayer ion conductance continued somewhat even after the voltage polarity was reversed to –70 mV (step 4), indicating that after an initial voltage-dependent initiation step, protein insertion into the bilayer became less voltage-dependent. APOL4 also caused a linear and continuous increase in ion conductance at +50 mV (step 1), but this increase became exponential after stepping to +70 mV (step 3). Unlike APOL3, this continuous increase in ion conductance completely stopped upon reversal of the voltage polarity from positive to negative (step 2), resulting in a constant ion conductance for as long as the negative voltage was maintained (Fig. 7*B*). Thus, the insertion of APOL4 into the bilayer to form an ion conductance was strictly voltage-dependent, occurring only at positive voltage across the bilayer. Like APOL4, APOL5 also inserted into the bilayer to form an ion conductance at neutral pH with the application of a positive voltage (step 1), and there was no further increase in bilayer ion conductance after switching to negative voltage (step 2) (Fig. 7*C*). To conclude, unlike APOL1 and APOL2, the insertion of APOL3, APOL4, and APOL5 into the bilayer to form an ion conductance occurred at neutral pH and in a voltage-dependent manner.

### APOL6 forms large unstable pores and causes membrane breakage

**Figure 8:**
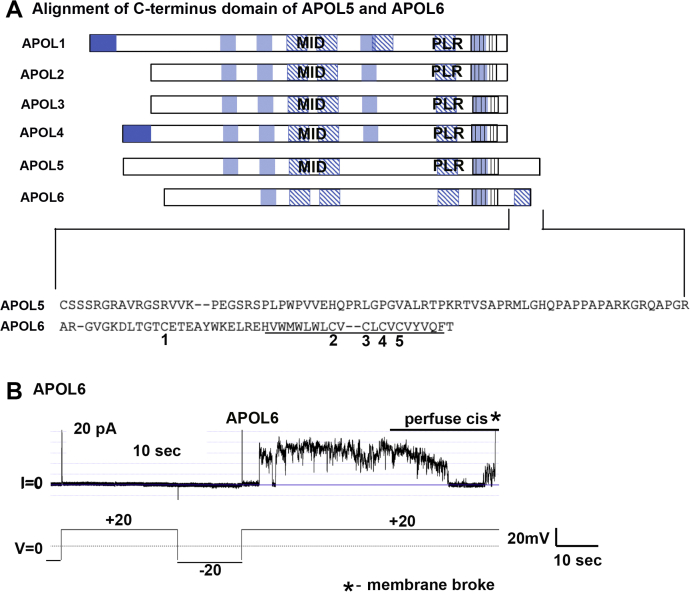
**APOL6 forms large unstable pores that were resolved by their dissolution or membrane breakage.***A*, *above*, domain structures of APOL1-6. *Below*, amino acid alignment of the APOL5 and APOL6 C-terminal extension that is absent in other APOLs. APOL6 has a predicted cysteine-rich (cysteines numbered below) transmembrane domain (underlined). *B*, a planar lipid bilayer was formed between symmetric solutions of bilayer buffer, pH 7.2. The current was recorded (upper trace) as the voltage was determined by the experimenter (lower trace). APOL6 (120 ng, 3.2 nmol) was added to the *cis* side prior to recording. Addition of APOL6 was consistently followed by apparent membrane instability and membrane breakage (representative trace shown; note that current trace goes off scale upon membrane breakage, as indicated by an *asterisk*).

Although the domain structure of APOL6 is somewhat similar to that of APOL3-5 (Fig. 1*B*), APOL6, unlike the other APOL proteins, has an extended C-terminus that includes a predicted transmembrane region that is rich in cysteine residues (Fig. 8*A*). Interestingly, APOL6 rapidly destabilized the bilayer membrane, resulting in membrane breakage that was sometimes preceded by the formation of large unstable pores (Fig. 8*B*), suggesting that the APOL6 C-terminus may interfere with the potential APOL6 ion-channel forming function.

### After inserting into the membrane, APOL1–APOL4 all generate cation-selective, pH-dependent ion permeation

**Figure 9:**
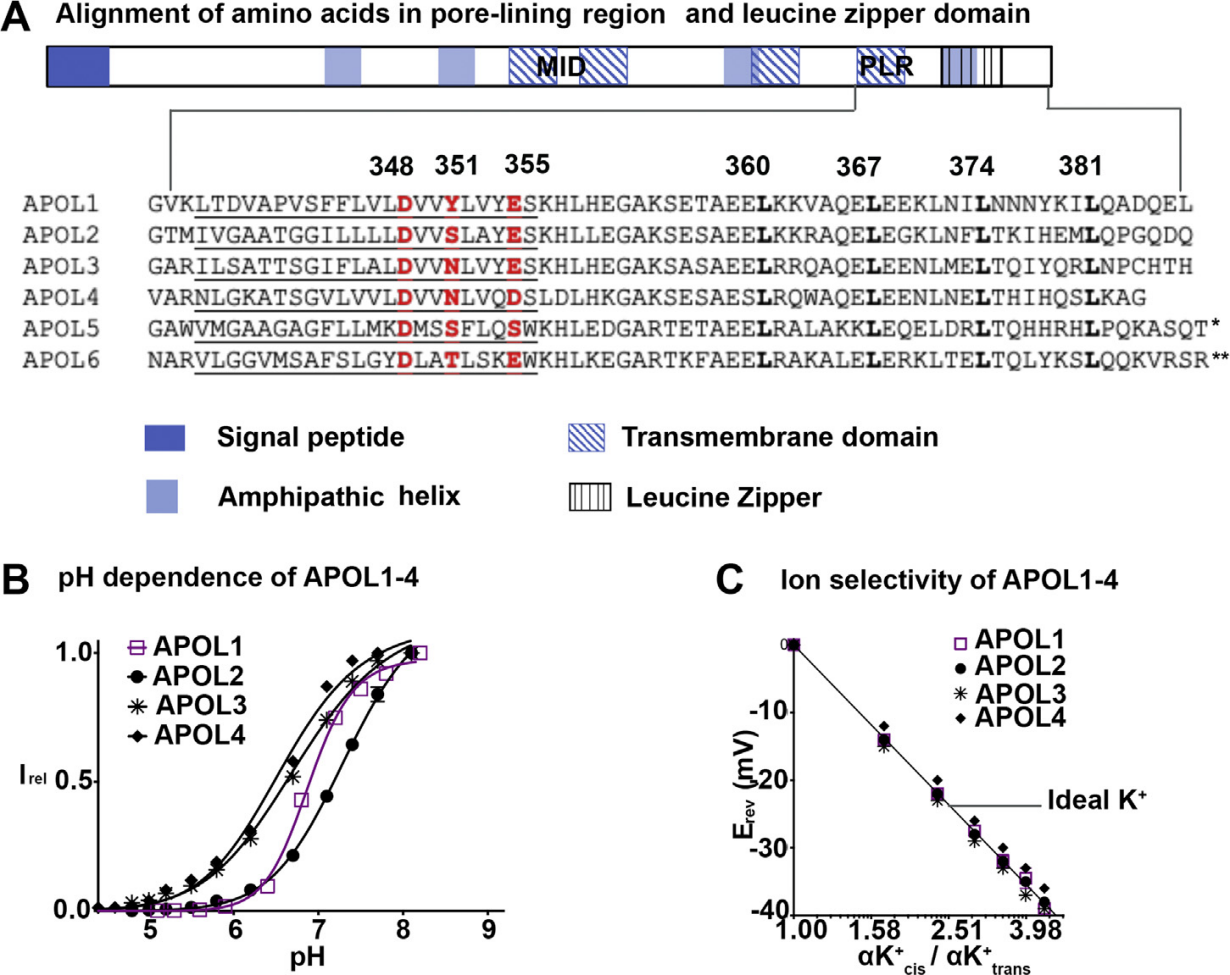
**APOL1-APOL4 form a pH-dependent cation-selective ion conductance.***A*, *above*, domain structure of APOL1. Below, amino acid alignment of APOL1-6 putative pore-lining region (PLR) and leucine zipper domains. The heptad-repeat leucine residues (leucine zipper domain) and a PLR residue that is critical to APOL1 pH-dependent cation channel activity (D, aspartic acid 348) are conserved throughout APOL1-6. ∗APOL5 has 76 more amino acids at the C-terminus, ∗∗APOL6 has 45 more amino acids at the C-terminus (*B*). A macroscopic bilayer ion conductance was formed at *cis* pH 5.6, *trans* pH 7.2 (APOL1 and 2), or at symmetric pH 7.2 (APOL3 and 4), as described in the legends to Figures 6 and 7. The pH was then adjusted to symmetric (*cis/trans*) pH 7.2 and the voltage was held at +20 mV to prevent further insertion of protein into the bilayer. The pH of both the *cis* and *trans* sides were then titrated in tandem with KOH and then back-titrated with HCl, keeping the voltage constant. Plotted is the steady-state current at each value of symmetric pH, relative to the maximal current (I_rel_). Results from a single representative experiment are shown, except in the case of APOL2, where average values ± SD from two independent experiments are plotted. *C*, a macroscopic bilayer conductance was obtained as above using APOL1-4 and then 3 M KCl was titrated into the *cis* compartment with symmetric pH 7.2 conditions. The reversal potential, or E_rev_ (voltage at which the registered current is zero) is plotted against the potassium activity gradient (αK^+^_cis_/αK^+^_*trans*_), which was determined as described in the Experimental procedures section. Results from a single representative experiment are shown, except in the case of APOL2, where average values ± SD from two independent experiments are plotted.

To characterize the macroscopic conductance (multiple ion channels) formed by the APOL proteins, in terms of pH dependence and ion selectivity, we focused on APOL1–4, due to difficulty purifying APOL5 and the tendency of APOL6 to destabilize the bilayer. In the case of APOL1, Schaub *et. al* showed that three amino acids in the fourth transmembrane domain (glutamic acid E355, tyrosine Y351, and aspartic acid D348) are in the PLR of the protein, affecting the pH gating and cation *versus* anion selectivity of the APOL1 channel (27), (Fig. 9*A*). Both pH gating and cation selectivity functions are critically dependent on the protonation status of the negatively charged residue aspartic acid 348 (D348), which is notably conserved throughout all the APOL proteins (Fig. 9*A*). Therefore, after obtaining a macroscopic conductance under the voltage/pH conditions favorable to the insertion of each APOL protein into the bilayer, we compared the pH dependence of the different APOL-induced ion conductance as the voltage was kept constant. As predicted from the conservation of the D348 side chain, each APOL protein produced an ion conductance that was sigmoidally dependent on pH, although the curve produced by APOL1 was characteristically steeper than that of the other proteins (Fig. 9*B*). In addition, the pKa (I_rel_ = 0.5) of the curves occurred within one pH unit of each other, at around neutral pH (Fig. 9*B*). We then compared the cation *versus* anion selectivity of the macroscopic ion conductance. As predicted from the conservation of the D348 side chain, all proteins were highly selective for potassium over chloride at constant pH 7.2 (Fig. 9*C*). Thus, while the APOL1–4 proteins differ in the conditions they require for membrane insertion, these results indicate that human APOL1–4 proteins all form pH-dependent cation-selective ion conductance and are active at neutral pH.

## Discussion

The *APOL1-6* gene family are innate immunity genes associated with antiviral activity (6, 7). In addition, APOL1 associates with HDL to form trypanosome lytic factor (TLF) complexes that provide innate immunity against many African trypanosome species and *Leishmania* sp (9, 14, 31). The *APOL* genes are rapidly evolving in the primate lineage, showing marks of positive selection due to a direct interaction with microbial pathogens (4). Although the actual role of *APOLs* is not known, some of the *APOL* genes are differentially expressed during diverse bacterial infections (24, 25). In addition, APOL1, APOL2, and APOL6 exhibit antiviral responses against various RNA viruses (6, 7). Based on these observations, APOL proteins are speculated to function in the innate immune response to a broad range of pathogens. However, very little is known about the precise biochemical function of the proteins in this gene family.

Although ostensibly similar in domain structure (Fig. 1*B*), we found that of the six human APOL proteins, only APOL1, APOL3, and APOL6 were cytotoxic when overexpressed in mammalian cells (Fig. 2). We show that APOL3 expression causes cytotoxicity and that the mode of cell death is similar to that of APOL1, accompanied by a swollen morphology of the cells (Fig. S3) (17, 20). Based on previous studies, in cells expressing APOL1 renal-risk variants G1 (S342G, I384M; rs73885319 and rs60910145) and G2 (del388:389NY, rs71785313), swelling and death were accompanied by sodium and calcium influx, as well as potassium efflux across the plasma membrane (17, 19). In contrast, cells dying of *APOL6* expression did not appear swollen (Fig. S3). Indeed, APOL6 overexpression has been shown to cause mitochondria-mediated apoptosis in cancer cell lines (23), and knockdown of APOL6 expression reduced interferon-induced apoptosis in atherosclerotic epithelial cells (32). Therefore, it is likely that APOL6 overexpression leads to apoptosis in HEK293 cells as well, a possibility that will require further investigation. We conclude that APOL1 and APOL3 proteins, which arose due to gene duplication, are more similar in function compared with the more distantly related and monophyletic APOL6 (Fig. 1).

APOL2, APOL4, and APOL5 were not cytotoxic when overexpressed, with no significant LDH release observed (Fig. 2, *B* and *C*). The low cytotoxicity of APOL4 and APOL5 could be explained by the reduced level of APOL4 and APOL5 proteins produced by the transfected cells (Fig. 2*D*). However, the level of APOL2 produced was the highest, despite it being noncytotoxic (Fig. 2, *C* and *D*). We also tested other naturally occurring variants of APOL1 and APOL4, which have differing signal peptide predictions (Fig. 3*A*). As reported previously (29), an APOL1 variant with a signal peptide (v. 1) was cytotoxic, while a variant with an unlikely signal peptide prediction was not (v. 3) (Fig. 3*B*). Our results confirm that the presence of a full signal peptide is required for APOL1-induced cytotoxicity, suggesting a role for APOL1 in the secretory pathway and in the ER. On the other hand, the presence of a predicted signal peptide did not affect the activity of APOL4 (Fig. 3*B*).

Previously, Schaub *et al*., showed that negatively charged glutamic acid residues in the APOL1 MID are critical for acid-dependent insertion of APOL1 into planar lipid bilayers (27). Both APOL1 and APOL2 have conserved negatively charged residues at the positions equivalent to E201 and E209 of the APOL1 MID, while all other APOLs have neutral amino acids substituted at the aligned positions (Fig. 5). As predicted, APOL2, like APOL1, inserts into lipid bilayers at acidic but not neutral pH (Fig. 6). Conversely, APOL3, APOL4, and APOL5, which have uncharged neutral amino acids aligning to APOL1 positions E201 and E209, were able to insert into lipid bilayers at neutral pH, providing additional evidence that these residues are critical for pH-dependent membrane insertion (Fig. 7). As previously observed in the case of bacterial pore-forming toxins (33), APOL3, 4, and 5 required a *cis*-positive voltage (the side to which the APOL proteins are added) to drive the formation of an ion conductance. Once formed, this conductance became largely independent of voltage (the ion conductance was maintained at negative voltage, Fig. 7), indicating that voltage is required for membrane insertion/channel assembly and does not affect ion permeation properties (channel gating or rectification). For example, a *cis* positive voltage could facilitate the translocation of positively charged amino acids (*i.e.*, lysine, arginine, and/or histidine residues) from the *cis* to the *trans* side of the bilayer during membrane insertion of APOL3, 4, and 5.

Next, we looked at the amino acids residues in APOL1-6 that align with the PLR (aa 335–355) of the APOL1 cation channel (27) (Fig. 9*A*). In particular, aspartic acid 348 (D348) is critical for pH gating and cation *versus* anion selectivity of the APOL1 ion channel. This aspartic acid is conserved throughout all APOL proteins, suggesting conservation of pH-dependent cation channel function. Because of the difficulty in purifying APOL5, and the membrane disruption due to APOL6, we tested the pH dependence and cation selectivity of APOL1-4 (Fig. 9*B*). As expected, the ion conductance formed by APOL1–4 proteins showed a sigmoidal dependence on pH, with pKa's occurring within one pH unit of each other and close to neutral pH. The pH dependence curve of APOL1 was steeper than that of the other APOLs tested, indicating an increased cooperativity of proton binding to more than one site. This may be due to the presence of a bulky tyrosine at position Y351 of APOL1 (which is absent from the other APOL proteins), because Y351 was previously found to couple the protonation of glutamic acid E355 to the protonation of aspartic acid D348 during APOL1 channel gating (27). As expected, the ion conductances formed by each APOL protein were highly selective for cations over anions at neutral pH (Fig. 9*C*) (13). These data suggest that APOL1–4 form cation conductances with similar ion permeation pathways.

The predicted domain structure of APOL6 is different from other APOLs in that it has a C-terminus extension that contains a cysteine-rich predicted transmembrane domain (Fig. 8*A*). Compared with the other APOL proteins, APOL6 behaved differently in membrane bilayers, causing membrane instability and disruption (Fig. 8*B*). This is in agreement with the previous evolutionary analysis of the *APOL* gene family where *APOL6* shows a distinct evolutionary pattern compared with other *APOLs* (4). The distinct cysteine-rich C-terminal domain of APOL6 is similar to the cysteine-rich domains in antimicrobial peptides, such as defensins and rattusin that disrupt lipid membrane integrity and permeabilize the membrane by a mechanism called the carpet model (34, 35). We hypothesize that the cysteine-rich C-terminus of APOL6 is responsible for the observed disruption of planar lipid bilayers (Fig. 8*B*), a possibility that should be further investigated. Previous analysis of the APOL6 protein for selection in human populations showed that the C-terminal extension is highly conserved (4), suggesting that it is critical for APOL6 function. It is therefore important to consider the location of affinity tags on the APOL proteins, as tagging the C-terminus of APOL6 may lead to changes in function.

Comparative analysis of the *APOL* gene family shows that *APOL5* and *APOL6* are the ancient genes in the family and have evolved separately from the *APOL1–4* gene cluster during primate evolution (4). Among the *APOL1–4* cluster, *APOL3* is considered most similar to the last common ancestor. Beginning approximately 33 million years ago, around the time of divergence of old-world monkeys and new-world monkeys, this gene led to the evolution of APOL1–4 genes (4, 5). Our results show that human APOL1–4 proteins slightly differ in their biochemical functions, whereas APOL6 is functionally distinct, in agreement with the gradual evolution of these proteins.

**Figure 10:**
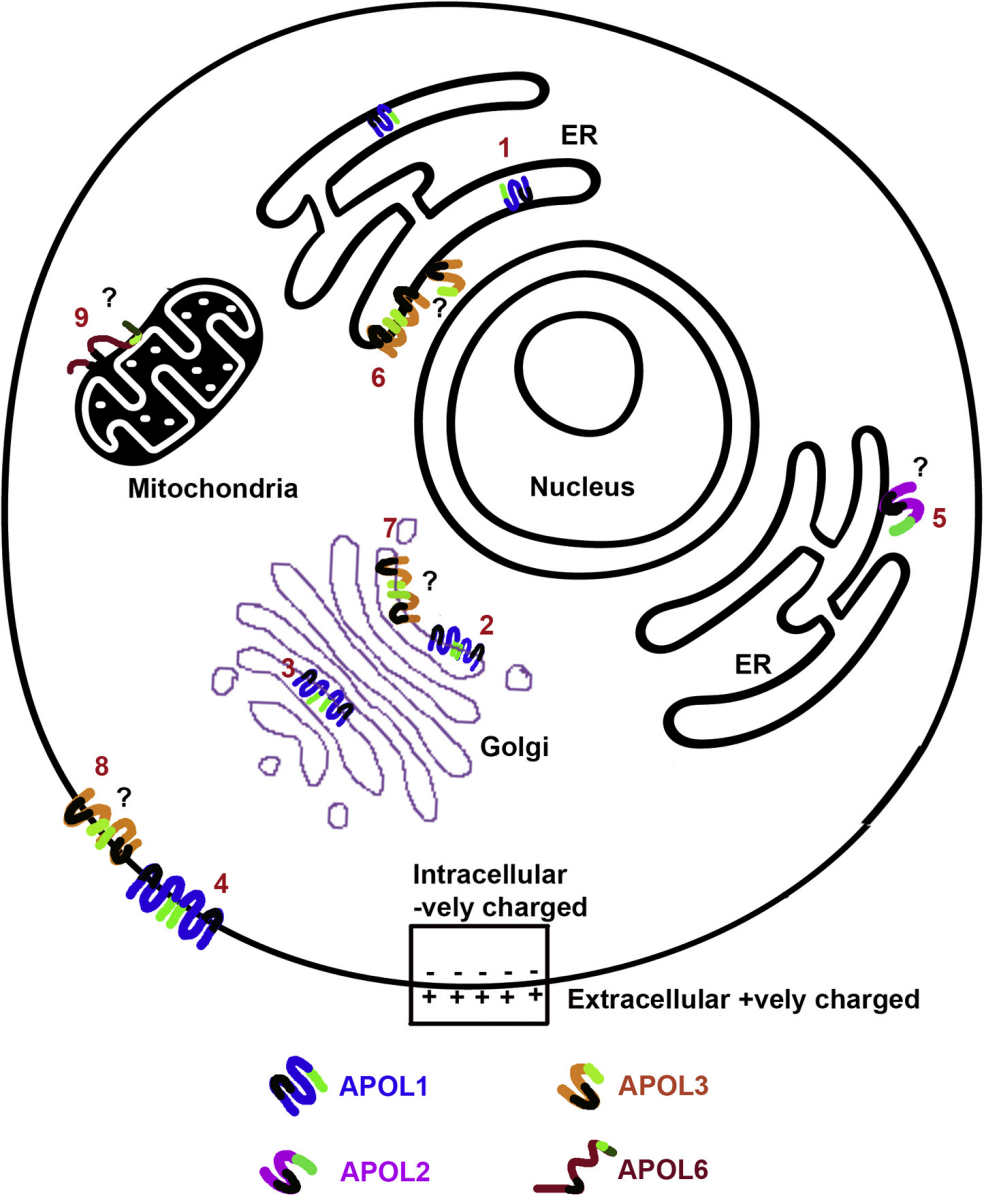
**A schematic of predicted subcellular localization of APOL proteins.** A schematic of predicted subcellular localization of the APOL protein family. The C-terminal leucine zipper domain is represented by the neon color except in APOL6, which has a dark green C-terminal transmembrane domain. The APOL1 (with signal peptide) is on the luminal side of ER (1) and Golgi apparatus (2) and associates with the membrane of these subcellular organelles (19, 30, 61). During its passage through the secretory pathway, APOL1 inserts into membranes at acidic pH (*e.g.*, in the *trans* Golgi, 3) to form closed nonselective cation channels that open in the plasma membrane upon exposure to neutral pH (4). APOL2 associates with the ER membrane on the cytoplasmic face (5) (30) and hence does not experience the acidic pH necessary for it to form ion channels. APOL3 associates with the ER (6) and Golgi membranes (7) on the cytoplasmic face (22) and possibly plasma membrane (8) to form ion channels. We do not know the localization of APOL4 and APOL5. Endogenous APOL6, which causes apoptosis, has a C-terminal transmembrane domain similar to apoptotic proteins, could potentially associate with the mitochondrial membrane (9) for its function (23). Except for APOL1, all other APOLs are hypothetically represented and hence may not be in the correct orientation. (Summarized in Table 1)

It is worth considering how these functional differences between APOL proteins could impact the different cellular membranes targeted by each protein. With regard to APOL1, we showed that the absence of an intact signal peptide in the naturally occurring variant APOL1 v.3. resulted in loss of cytotoxicity when overexpressed in human cells (Fig. 3*B*), as previously reported (18, 29). We attribute this loss of cytotoxicity to the reported decrease in ER lumenal localization of APOL1 v.3 compared with APOL1 v.1 (30). The cytotoxicity due to APOL1 v.1 renal-risk variants was dependent on APOL1 release from the ER and was associated with sodium and calcium influx across the plasma membrane (19). These data support a model in which APOL1-induced cytotoxicity results from APOL1 channel formation during transit through an acidic post-ER compartment, such as the *trans* Golgi and/or secretory vesicles, followed by cation channel opening at neutral pH in the plasma membrane (Fig. 10 and Table 1). Notably, we found that like APOL1, the development of a full cation conductance by APOL2 required an acid dependent activation (insertion and dimerization) step, followed by pH neutralization (Fig. 6). However, unlike APOL1, APOL2 was not cytotoxic when overexpressed in human cells, despite a high level of protein production (Fig. 2). As is the case with APOL1 v.3, the loss of cytotoxicity may result from the absence of a signal peptide in APOL2, consistent with the reported localization of APOL2 to the cytoplasmic face of the endoplasmic reticulum (Fig. 10 and Table 1) similar to APOL1 v.3 (30). The fact that *APOL2* is full length and show marks of positive selection in primate lineage supports the hypothesis that APOL2 is functional and is an innate immunity protein. However, it can form an ion conductance only after activation and membrane insertion at an acidic pH. One phenomenon used by host cells to defend against viral infection is autophagy, which creates double membrane autophagosomes derived from ER membranes and delivers viral cargo to lysosomes for degradation (36, 37). Therefore, it is possible that APOL2 and APOL1 v.3 associated with the cytoplasmic face of the endoplasmic reticulum are involved in antiviral infection where the proteins can get activated in the acidic environment of mature autophagosomes and autolysosomes (38, 39).

**Table 1:** Succinct summary of key findings/features

Proteins	pH/voltage requirements		Cytotoxic in overexpressing cells	Protein production detected by western blot	Predicted membranes for APOLs channel formation
	Insertion into membrane	Ion permeation			
APOL1 with signal peptide	Acidic pH on the *cis* (lumenal) and neutral pH on the *trans* (cytosolic) side	Neutral pH on the *cis* (extracellular) side and neutral pH on the *trans* (cytosolic) side	Yes. Cells appear swollen with LDH release.	+++	Membranes of exocytic compartment where the protein can insert into the membrane at acidic pH in Golgi stacks and form channels in the plasma membrane when the membrane inserted protein is exposed to neutral pH (13, 19, 30)
APOL2	Acidic pH on the *cis* (lumenal) and neutral pH on the *trans* (cytosolic) side	Neutral pH on the *cis* side and neutral pH on the *trans* side	No	++++	Acidic interior of autophagic vacuole
APOL3	*cis* (cytosolic)- positive voltage	Neutral pH on the *cis* (cytosolic) side and neutral pH on the *trans* (lumenal/extracellular) side	Yes. Cells appear swollen with LDH release.	+++	ER, Golgi (22), and plasma membrane
APOL4	*cis* (cytosolic)- positive voltage	Neutral pH on the *cis* (cytosolic) side and neutral pH on the *trans* (lumenal/extracellular) side	No	+	Cytoplasmic face of ER?
APOL5	*cis* (cytosolic)- positive voltage	Neutral pH on the *cis* (cytosolic) side and neutral pH on the *trans* (lumenal/extracellular) side	No	+	Cytoplasmic face of ER?
APOL6			Yes. Cells release LDH. No swollen cells observed.	+	Mitochondria (23)

In contrast to APOL1 and APOL2, we found that APOL3, APOL4, and APOL5 did not require an acidic pH in order to generate a bilayer ion conductance, potentially allowing these proteins (which lack a signal peptide) to insert into cellular membranes from the cytoplasmic face to form a cation-selective conductance (Fig. 7). However, initiation of an ion conductance (membrane insertion and dimerization) in each case was dependent on the presence of a *cis* (*i.e.*, cytoplasmic face) positive voltage/membrane potential. Given the osmotic swelling that was apparent after APOL3 overexpression, APOL3-induced cytotoxicity could result from the eventual formation of a cation conductance in the plasma membrane (Fig. 10 and Table 1). However, we do not know conditions under which APOL3 inserts into plasma membrane. One possibility could be *via* membrane contact sites (40). Although the resting membrane potential (inside –80 mV) across the plasma membrane would seem to prohibit APOL4 and APOL5 insertion into the plasma membrane, conductance formation by APOL3 was less strictly voltage-dependent than APOL4 (Fig. 7). Thus, preferential insertion of APOL3 into the membrane, along with reduced protein levels of APOL4 and APOL5 compared with APOL3, may account for the cytotoxicity we observed with APOL3 but not APOL4 or APOL5 production (Fig. 2). Alternatively, APOL3 was previously found to localize to the ER of primary human podocytes, with partial Golgi localization (Fig. 10 and Table 1), which was attributed to the observed affinity of APOL3 for the anionic phospholipids found in these organelles (22). Whether the membrane potential across the Golgi or ER membranes is appropriate to drive voltage-dependent membrane insertion by APOL3 is not clear, due to difficulties in measuring the membrane potentials of internal membranes. However, based on one analysis using a voltage reporter that appeared to partition between the ER and plasma membranes (41), the ER membrane potential was found to be positive on the cytoplasmic face and thus amenable to APOL3 insertion from the cytoplasmic face.

Finally, the monophyletic APOL6 has a different domain structure than all other APOLs, with a C-terminal extension that contains a cysteine-rich predicted transmembrane domain. APOL6 overexpression leads to apoptosis, which was associated with cytochrome-c and Smac/DIABLO release from the mitochondria of human cancer cells (23). It will be worth exploring whether the C-terminal transmembrane of APOL6 is a mitochondrial targeting sequence, analogous to the C-terminal transmembrane of various proteins of the Bcl-2 family of apoptosis regulators (42). However, unlike that of Bcl-2 family proteins, the C-terminus of APOL6 also resembles the cysteine-rich motif of antimicrobial peptides that leads to membrane disruption (34, 35). Therefore, the C-terminal region of APOL6 is likely to play a vital role in the localization and function of the protein. It also raises the possibility that APOL6 has broad spectrum antimicrobial activity, a possibility that should be studied in the future.

Our study shows that APOL proteins, although structurally similar, differ in their biochemical activities and likely target membranes, suggesting that they may have evolved to combat a range of different microbial pathogens. APOL1 has been shown to exert antiviral activity against Human Immunodeficiency virus (HIV1) and other RNA viruses including flavivirus, togavirus, and paramyxovirus (6, 7, 43). APOL2 was shown to inhibit Hepatitis C virus infection and APOL6 has been shown to inhibit poliovirus (6, 7). In addition, at least three studies have shown expression of *APOL1* and *APOL6* genes change in response to infection by the intracellular bacterial pathogens *Mycobacterium* and *Listeria* (24, 25, 44). It is likely that these APOLs provide immunity against broader range of pathogens or are involved in different pathways of innate immunity, which should be studied in the future. The mechanism by which these APOLs exert antimicrobial activities has not yet been extensively studied, although the antiparasitic role of APOL1 against trypanosomes and leishmania is well established (9, 13, 14, 15, 45). APOL1, as part of the TLF complex, directly interacts with kinetoplastid parasites trypanosome and leishmania and causes swelling and lysis of these parasites (9, 14, 15, 45). APOL1 has also been shown to restrict HIV infection in transiently transfected HEK293 cells (43). Whether other APOL proteins directly interact with different microbes to inhibit microbial infection has yet to be investigated.

The *APOL* genes belong to the interferon regulated genes (IRGs) and are upregulated by various inflammatory signals including interferons, tumor necrosis factor alpha, and viral mimetics (6, 25, 46, 47) supporting their role in innate immunity. Inflammatory signals such as interferons and tumor necrosis factor alpha can induce cellular death by apoptosis and necroptosis pathways (48, 49), thereby inhibiting the spread of the influenza viruses. Therefore, it is possible that APOL1, APOL3, and APOL6 operate downstream of interferon signaling to induce cytotoxicity and cell death and thereby prevent viral growth and spreading. Moreover, viruses use cellular membranes such as plasma membranes, ER, Golgi, and mitochondrial membranes for entry, replication, and growth in the cells (50, 51, 52). Hence, APOLs associated with various membranes such as the plasma membrane, ER, Golgi, and mitochondria could indirectly affect viral replication and growth (Fig. 10). Many viruses produce ion-channel proteins to manipulate various intracellular membranes for their replication and growth, and hence these channels have been considered as a potential drug target for various viral infections (52). Therefore, APOLs functioning as ion channel-forming proteins could affect viral replication. Finally, different immune cells such as T-cells and macrophages use cytoplasmic calcium entry due to various ion channel proteins as a signal for their downstream effector function. Hence it is possible that the cation channel activity of APOL1-5 could regulate immune cell function. The potential roles of different APOL proteins in the innate immune system should be further investigated in the future.

## Experimental procedures

### Protein structure prediction

The protein sequences used for structural prediction are given in Table S1. The signal peptide prediction was done using prediction software SignalP-5.0 server (28). To predict amphipathic helices, we used previously published data (3) that was validated using prediction software HeliQuest (53). The transmembrane regions were predicted using TMPRED (54) and Phobius (55, 56) and the Leucine zipper domain was predicted using coiled-coil prediction software (57).

### Generation of tagged APOL expression constructs

APOL2-6 coding sequences were generously gifted by Dr Sarah Garrett and Dr Steve Almo of the Albert Einstein College of Medicine (see Table S1 for encoded amino acid sequences and accession numbers). To allow for bacterial production of recombinant APOL proteins, each coding sequence was then subcloned by PCR and ligation-independent cloning into the pNIC28-BSAI bacterial expression vector (Addgene), such that a vector-encoded 6× Histidine-tag was appended in-frame to the N-terminus of each coding sequence. In the case of APOL4, the sequence encoding the predicted signal peptide was excluded (Fig. 3*A*). The most globally common APOL1 G0 variant (Table S1) was cloned into pNIC28 without a signal peptide as previously reported (13). The sequence of the entire open reading frame was then checked to ensure no errors had been introduced.

To allow for protein production in mammalian cells, we used the APOL1 G0 sequence (Table S1) inserted into pRG977 as described previously (15, 58). In this vector, gene expression is controlled by the ubiquitin promoter and transcripts are stabilized by a beta-globin intron. For APOL2-6 expression we used cDNAs encoding the same amino acid sequences listed in Table S1. These coding sequences were amplified by PCR using the primer sequences listed in Table S2 and inserted into pRG977 between XbaI and EcoRI restriction enzyme cut sites. In the case of Myc-tagged constructs, G-block fragments were designed. Each G-block sequence contains 5′ XhoI and 3′ XbaI sites for restriction enzyme-based insertion into the mammalian expression vector pRG977. The Myc-tag followed by 2xGGSGG linker was placed immediately downstream of Kozak and start sequences and punctuated by the XbaI site. The APOLs were amplified with primers (Table S2) containing 5′ XbaI and 3′ EcoRI restriction sites for insertion into myc-tag containing PRG977. For APOL1, the signal peptide was placed immediately upstream of the myc tag, and the APOL1 sequence downstream of the signal peptide cleavage site was inserted *via* the XbaI and EcoRI sites.

### Expression in mice and functional assay of Myc tagged APOL1

In total, 50ug of *Myc:APOL1*, *APOL1* or pRG977 (Empty vector) was injected into mice (Swiss Webster, Taconic) *via* tail vein by hydrodynamic gene delivery to create transiently transfected mice.

A day after transfection, when the transfected genes were expressed, the mice were intraperitoneally injected with 5000 Lister-derived *T. b. brucei.* 427 African trypanosomes. Infected mice were monitored for parasitemia and survival for 35 days after infection. All animal experiments were approved by the Hunter College Institutional Animal Care and Use Committee (IACUC); animal welfare assurance agreement number D16-00413 (A3705-01) from the National Institute of Health, Office of Laboratory Animal Welfare.

### Expression in cell lines and cytotoxicity assay

HEK293 cells were grown in DMEM (Corning, 10-013-CV) containing 10% fetal bovine serum. For consistency, cells grown between passages 2 and 8 after thawing from liquid nitrogen were used. Cells were seeded at 1 × 10^4^ cells per well, in a 96-well plate, and then transfected with 100 ng of plasmid DNA using lipofectamine 3000 (Invitrogen, L3000001) according to manufacturer's protocol. After 6 h of transfection, cells were changed into fresh media.

For cytotoxicity assays, media samples were collected at different time points as indicated and were centrifuged to 8000*g*. The supernatants were collected and used to measure lactate dehydrogenase using CytoTox 96 Non-Radioactive Cytotoxicity Assay (Promega, G1780) following the manufacturer protocol. The percent of LDH released was normalized to nontransfected cells lysed (using lysis buffer according to manufacturer protocol) for an hour (as 100% LDH released) and nontransfected cells not lysed (as 0% LDH released).

To test for protein expression, cells were washed twice with PBS and then lysed directly into the Laemmli buffer (Bio-Rad, 161-0737) with 5% beta mercaptoethanol. The lysed samples were run on an SDS PAGE gel (Bio-Rad, 4561033 and 4561036) to separate proteins based on their size and then immunoblotted by probing for APOLs using rabbit anti-Myc antibody (Proteintech, 16286-1-AP, 1:10,000) and anti-Rabbit IgG HRP (Rockland Inc, 18-8816-33, 1:5000); and for GAPDH using rabbit anti-GAPDH antibody (Proteintech, 10494-1-AP, 1:10,000) and anti-rabbit IgG HRP antibody (Promega, W4011, 1:5000).

### Recombinant protein production and purification

Recombinant APOL proteins were produced in *E. coli* Bl21-DE3-RIPL cells and purified from inclusion bodies as previously published for APOL1 (27), but with minor modifications to increase stability and solubility of the purified proteins. Briefly, cells were transformed with the expression vector and on the following day colonies were directly inoculated into overnight express-TB auto-induction medium and grown with vigorous shaking (300 rpm) for 16 h. Cells were lysed by resuspension in hypotonic lysis buffer (50 mM Tris-HCl pH 8.0, 1 mM EDTA), which was supplemented just before use with 0.5 mM DTT and the irreversible serine protease inhibitor 4-(2-aminoethyl) benzene sulfonyl fluoride hydrochloride (AEBSF). Inclusion bodies were then isolated as described previously (27) except that 0.5 mM DTT was maintained throughout the process to maintain a reducing environment and HALT protease inhibitor cocktail (Thermo Fisher, Cat# 78429) was added in all steps to prevent proteolytic degradation of proteins. Proteins were then solubilized in the detergent Zwittergent 3-14 and purified by nickel-affinity chromatography (His-TRAP, 1 ml; GE), followed by size-exclusion chromatography (SEC; Superdex-200, GE). The SEC column was equilibrated in SEC buffer (50 mM Tris-HCl [pH 7.4], 150 mM NaCl, 0.05% zwittergent 3-14, 0.5 mM DTT) and then the proteins were injected onto the column and eluted in the same buffer. The protein products were checked for purity by SDS PAGE, followed by staining with Coomassie Blue R-250 (Fig. 4). All proteins were flash-frozen in SEC buffer, on dry ice, and stored in aliquots at –80 °C.

### Planar lipid bilayer assays

Planar lipid bilayers were generated from the union of two lipid monolayers across a 150–200 micron hole in a 50 micron thick Teflon partition, as described previously (13). Briefly, the Teflon partition was pretreated with a 3% squalene solution in petroleum ether and then a chamber buffer (150 mM KCl, 5 mM CaCl2, 0.5 mM EDTA, 5 mM K-MES, 5 mM K-HEPES, pH 7.2) was added to compartments on either side. Twenty microliters of a 1.5% (w/v) asolectin (soybean phospholipids from which nonpolar lipids had been removed) (59), 0.5% (w/v) cholesterol solution in pentane was then layered on top of each aqueous solution and the solvent was allowed to evaporate. The solutions on each side were then connected to a pair of silver/silver chloride electrodes *via* salt bridges. Connected to the electrodes was a voltage-clamp amplifier (Warner instruments BC535), which was used to apply voltage and measure the current output. Lipid monolayers were brought above the hole on each side and bilayer formation was detected as an increase in system capacitance in response to a triangle wave of voltage. The solutions on both sides were stirred using magnetic stirrers and the pH was adjusted with the addition of precalibrated volumes of 0.5 M HCl or 0.5 M KOH as appropriate. The *cis* side refers to the side to which protein was first added and the voltage is reported as that of the *cis* side relative to the *trans*. The reversal potential (voltage at which current reverses the direction) was determined by adjusting the voltage until the macroscopic conductance read zero. Ideal cation selectivity was calculated using the Nernst potential at various ratios of *cis*:*trans* KCl activities. Activity coefficients were obtained from appendix 8.10, table 11 of reference (60), where we took the KCl concentration to be equal to the K^+^ concentration.

## Data availability

All relevant data are contained in the manuscript or in the supplementary information.

## Supporting information

This article contains supporting information.

## Conflict of interest

The authors declare that they have no conflicts of interest with the contents of this article.
