# Supplementary material for: Apolipoproteins L1-6 share key cation channel-regulating residues but have different membrane insertion and ion conductance properties
Source: J Biol Chem. 2021 Jul 10;297(2):100951. doi: 10.1016/j.jbc.2021.100951 (PMC8358165; doi:10.1016/j.jbc.2021.100951)
Supplement: Figures S1–S3 and Tables S1 and S2 [file mmc1.pdf]

Apolipoproteins L1-6 share key cation channel-regulating residues but have different membrane insertion and ion conductance properties

Jyoti Pant*[1], Joseph A Giovinazzo[1, 3], Lilit S., Tuka[1], Darwin Pena[1], Jayne Raper[1, 2] and Russell Thomson*[1]

[1]Department of Biological Sciences, Hunter College, CUNY, New York, USA

[2]Department of Biochemistry, The graduate Center, CUNY, New York, USA

[3]Now at Department of Biochemistry and Molecular Genetics, School of Medicine, University of Colorado Anschutz Medical Campus, Aurora, CO, USA

* Corresponding authors – Jyoti Pant and Russell Thomson

Email- rthomson@genectr.hunter.cuny.edu

jpanta@genectr.hunter.cuny.edu

Running Title: Functional Characterization of Apolipoproteins L Family

Supplementary Figures 1-3 and Supplementary Table 1 and 2

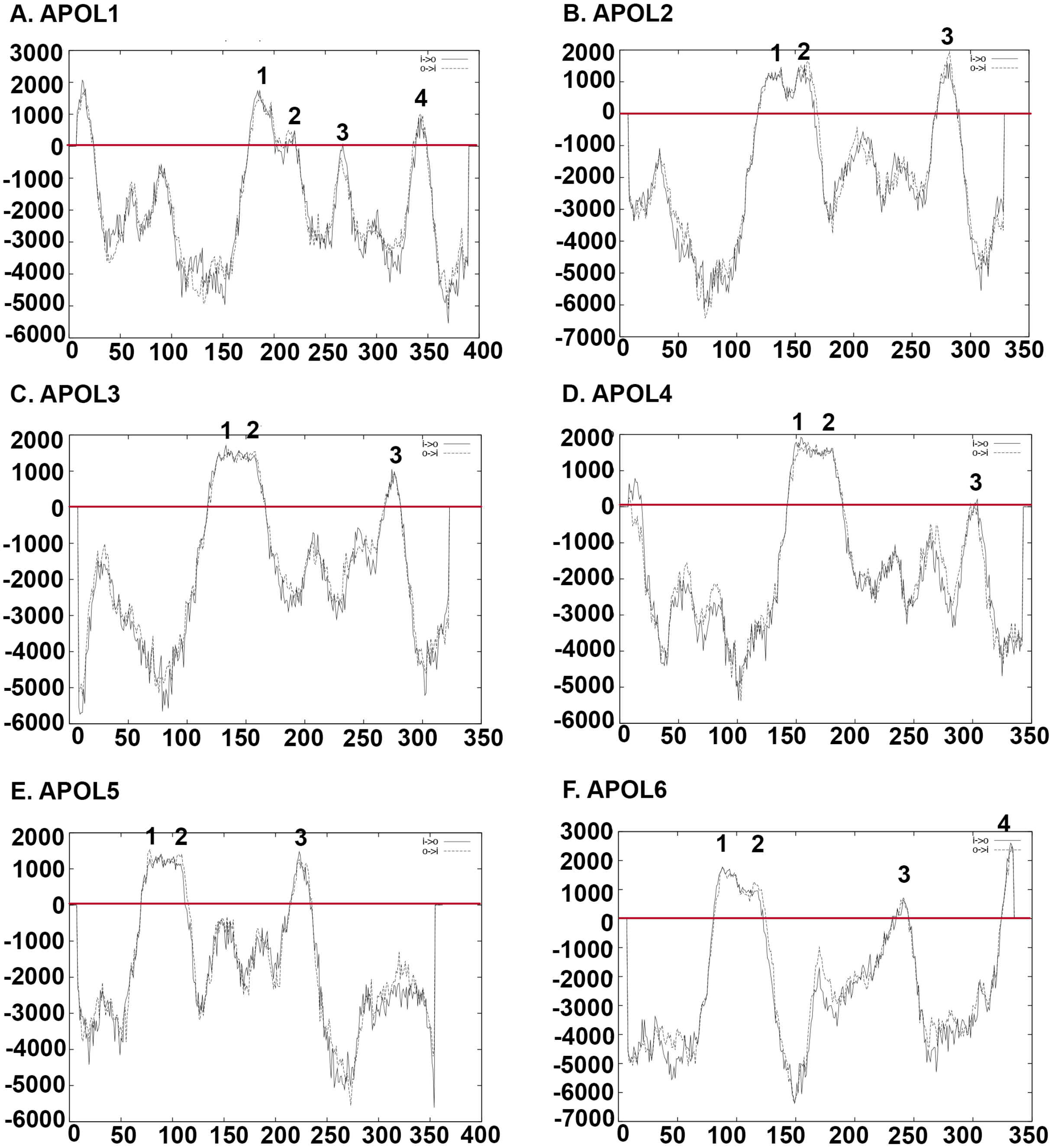
A. APOL1
B. APOL2
C. APOL3
D. APOL4
E. APOL5
F. APOL6
i->o
o->i
1
2
3
4

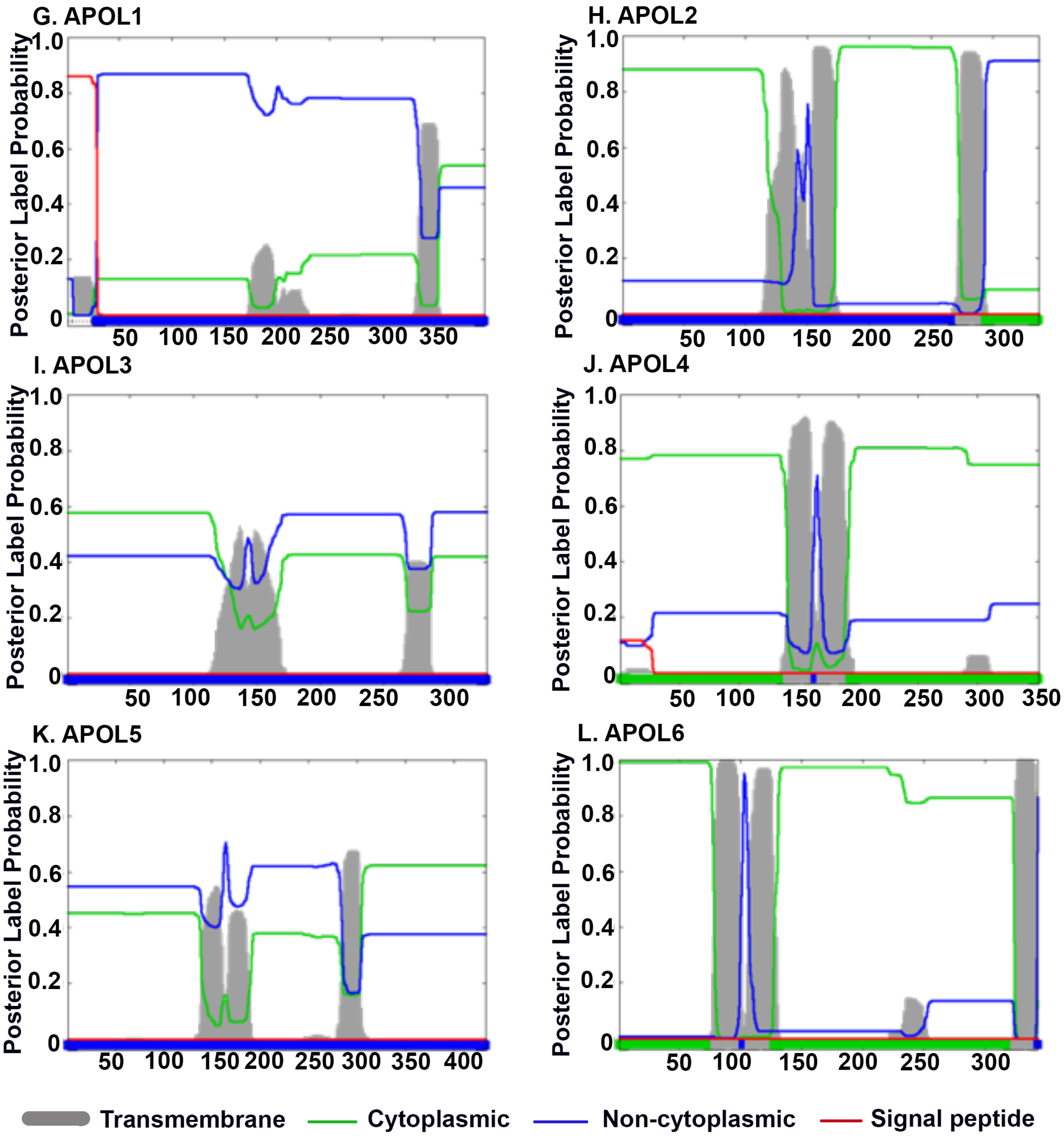
G. APOL1
H. APOL2
I. APOL3
J. APOL4
K. APOL5
L. APOL6
Posterior Label Probability
1.0
0.8
0.6
0.4
0.2
0
50 100 150 200 250 300 350
50 100 150 200 250 300
50 100 150 200 250 300
50 100 150 200 250 300 350
50 100 150 200 250 300 350 400
50 100 150 200 250 300
Transmembrane
Cytoplasmic
Non-cytoplasmic
Signal peptide

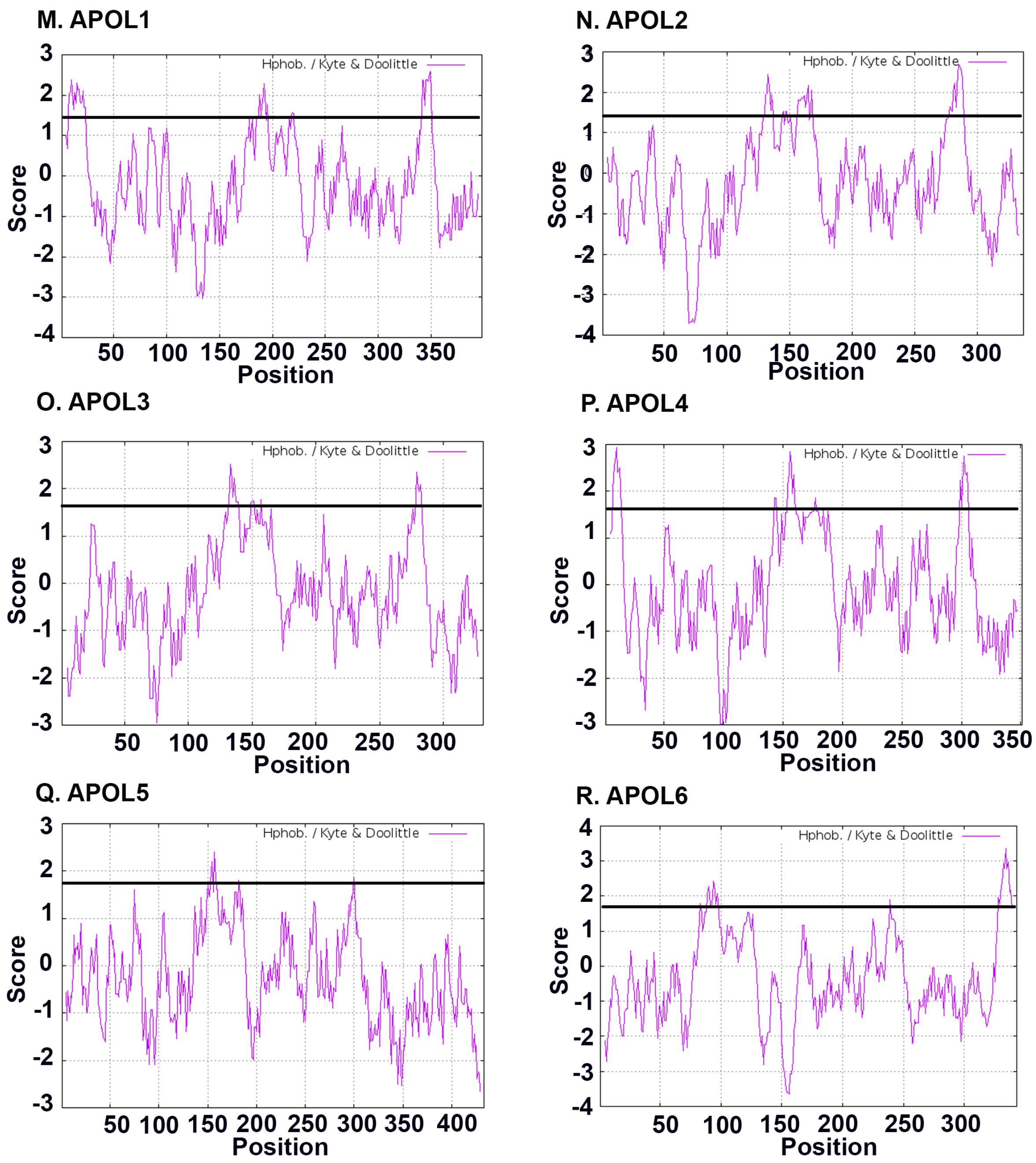

Supplementary Figure 1

(A-F) Predicted transmembrane regions of APOL1-6 using TMpred (1). (G-L) Predicted transmembrane regions of APOL1-6 using Phobius (2,3) . (I-R) Predicted hydropathy plot using Kyte-Doolittle scale (4).

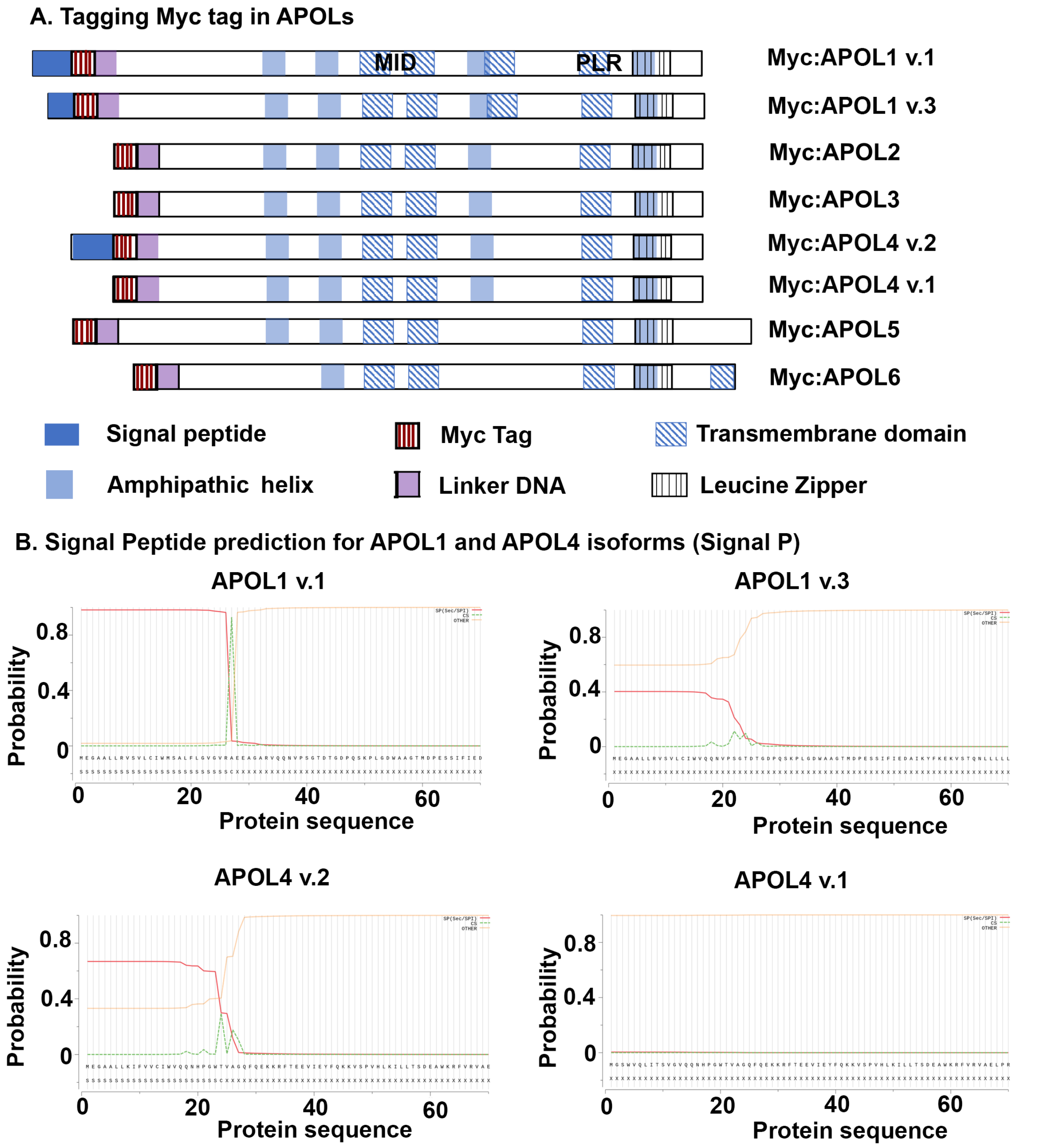

Supplementary Figure 2

(A) Myc tags were appended to the N-terminal coding sequences of APOL1-APOL6 via a 10 amino acid linker. as indicated (B) Prediction for signal peptide for APOL1v.1, APOL1 v.3, APOL4 v.1 and APOL4 v.2 using SignalP-5.0 (5).

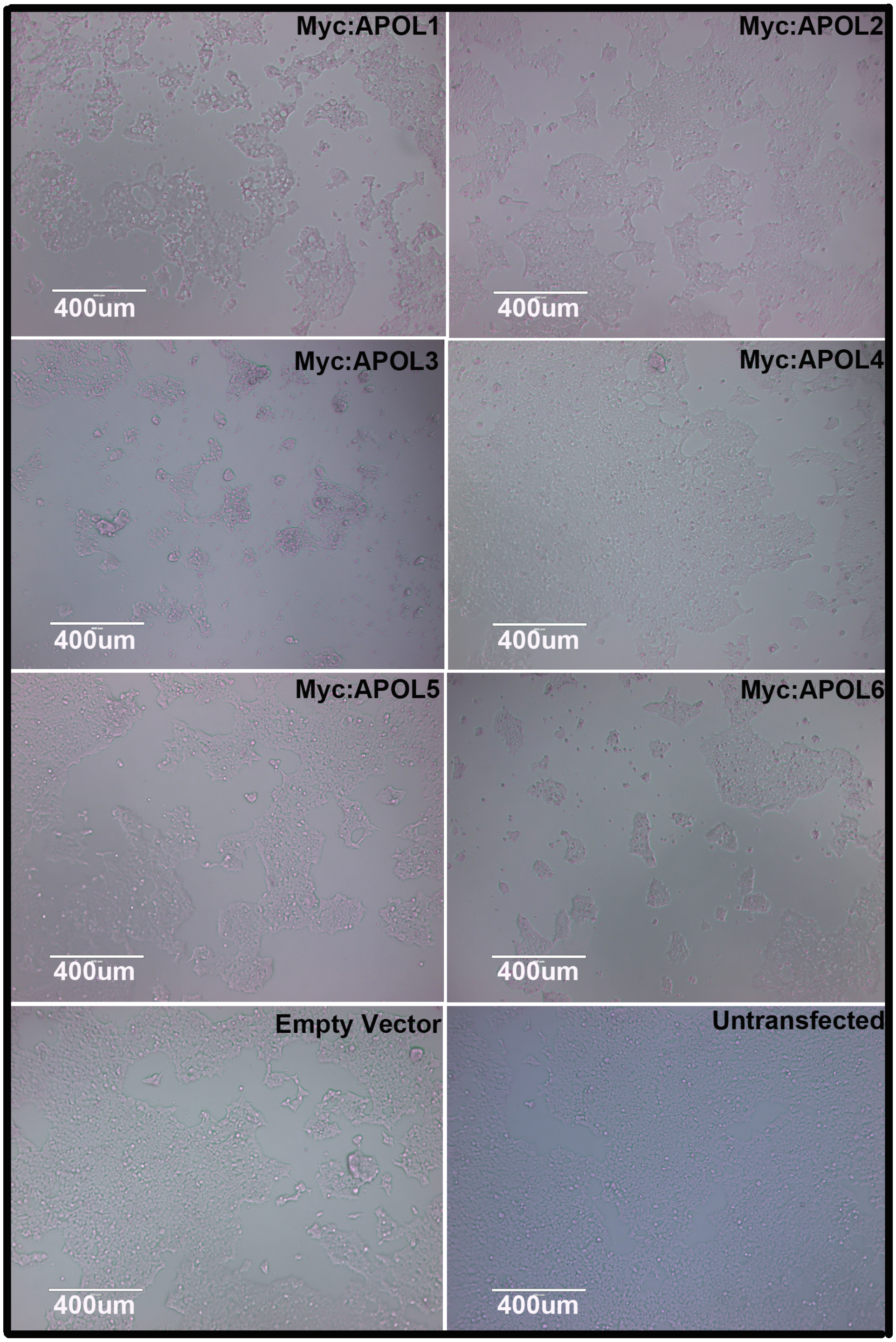

Supplementary Figure 3

Hek 293 cells were transfected using 100 ng of plasmid DNA containing Myc-tagged *APOL1-APOL6.* Morphology of HEK 293 cells were recorded 24 hours post transfection using a Evos light microscope under 10X magnification (scale bar 400um).

Supplementary Table 1.

**APOL1 isoform 1 (GenBank: AAI12944.2)**<br>MEGAALLRVSVLCIWMSALFLGVGVRAEEAGARVQQNVPSGTDTGDPQSKPLGDW<br>AAGTMDPESSIFIEDAIKYFKEKVSTQNLLLLLTDNEAWNGFVAAAELPRNEADELRK<br>ALDNLARQMIMKDKNWHDKGQQYRNWFLKEFPRLKSKLEDNIRRLRALADGVQKV<br>HKGTTIANVVSGSLSISSGILTLVGMGLAPFTEGGSLVLLEPGMELGITAALTGITSSTI<br>DYGKKWWTQAQAHDLVIKSLDKLKEVKEFLGENISNFLSLAGNTYQLTRGIGKDIRAL<br>RRARANLQSVPHASASRPRVTEPISAESGEQVERVNEPSILEMSRGVKLTDVAPVSF<br>FLVLDVVYLVYESKHLHEGAKSETAEELKKVAQELEEKLNILNNNYKILQADQEL
**APOL1 isoform 3 (GenBank: BAG62174.1)**<br>MEGAALLRVSVLCIWVQQNVPSGTDTGDPQSKPLGDWAAGTMDPESSIFIEDAIKYF<br>KEKVSTQNLLLLLTDNEAWNGFVAAAELPRNEADELRKALDNLARQMIMKDKNWHD<br>KGQQYRNWFLKEFPRLKSKLEDNIRRLRALADGVQKVHKGTTIANVVSGSLSISSGIL<br>TLVGMGLAPFTEGGSLVLLEPGMELGITAALTGITSSTIDYGKKWWTQAQAHDLVIKS<br>LDKLKEVKEFLGENISNFLSLAGNTYQLTRGIGKDIRALRRARANLQSVPHASASRPR<br>VTEPISAESGEQVERVNEPSILEMSRGVKLTDVAPVSFFLVLDVVYLVYESKHLHEGA<br>KSETAEELKKVAQELEEKLNILNNNYKILQADQEL
**APOL2 (NP_112092.2)**<br>MNPESSIFIEDYLKYFQDQVSRENLLQLLTDDEAWNGFVAAAELPRDEADELRKALN<br>KLASHMVMKDKNRHDKDQQHRQWFLKEFPRLKRELEDHIRKLRALAEEVEQVHRG<br>TTIANVVSNSVGTTSGILTLLGLGLAPFTEGISFVLLDTGMGLGAAAAVAGITCSVVEL<br>VNKLRARAQARNLDQSGTNVAKVMKEFVGGNTPNVLTLVDNWYQVTQGIGRNIRAI<br>RRARANPQLGAYAPPPHVIGRISAEGGEQVERVVEGPAQAMSRGTMIVGAATGGILL<br>LLDVVSLAYESKHLLEGAKSESAEELKKRAQELEGKLNFLTKIHEMLQPGQDQ
**APOL3 (NP_055164.1)**<br>MDSEKKRFTEEATKYFRERVSPVHLQILLTNNEAWKRFVTAAELPRDEADALYEALK<br>KLRTYAAIEDEYVQQKDEQFREWFLKEFPQVKRKIQESIEKLRALANGIEEVHRGCTI<br>SNVVSSSTGAASGIMSLAGLVLAPFTAGTSLALTAAGVGLGAASAVTGITTSIVEHSY<br>TSSAEAEASRLTATSIDRLKVFKEVMRDITPNLLSLLNNYYEATQTIGSEIRAIRQARA<br>RARLPVTTWRISAGSGGQAERTIAGTTRAVSRGARILSATTSGIFLALDVVNLVYESK<br>HLHEGAKSASAEELRRQAQELEENLMELTQIYQRLNPCHTH
**APOL4 isoform 2 (NP_663693.1)**<br>MEGAALLKIFVVCIWVQQNHPGWTVAGQFQEKKRFTEEVIEYFQKKVSPVHLKILLTS<br>DEAWKRFVRVAELPREEADALYEALKNLTPYVAIEDKDMQQKEQQFREWFLKEFPQ<br>IRWKIQESIERLRVIANEIEKVHRGCVIANVVSGSTGILSVIGVMLAPFTAGLSLSITAAG<br>VGLGIASATAGIASSIVENTYTRSAELTASRLTATSTDQLEALRDILRDITPNVLSFALD<br>FDEATKMIANDVHTLRRSKATVGRPLIAWRYVPINVVETLRTRGAPTRIVRKVARNLG<br>KATSGVLVVLDVVNLVQDSLDLHKGAKSESAESLRQWAQELEENLNELTHIHQSLKA<br>G
**APOL4 isoform 1 (NP_001373814)**<br>MGSWVQLITSVGVQQNHPGWTVAGQFQEKKRFTEEVIEYFQKKVSPVHLKILLTSDE

AWKRFVRVAELPREEADALYEALKNLTPYVAIEDKDMQQKEQQFREWFLKEFPQIR<br>WKIQESIERLRVIANEIEKVHRGCVIANVVSGSTGILSVIGVMLAPFTAGLSLSITAAGV<br>GLGIASATAGIASSIVENTYTRSAELTASRLTATSTDQLEALRDILRDITPNVLSFALDF<br>DEATKMIANDVHTLRRSKATVGRPLIAWRYVPINVVETLRTRGAPTRIVRKVARNLGK<br>ATSGVLVVLDVVNLVQDSLDLHKGAKSESAESLRQWAQELEENLNELTHIHQSLKAG
**APOL5 (NP_085145.1)**<br>MPCGKQGNLQVPGSKVLPGLGEGCKEMWLRKVIYGGEVWGKSPEPEFPSLVNLCQ<br>SWKINNLMSTVHSDEAGMLSYFLFEELMRCDKDSMPDGNLSEEEKLFLSYFPLHKF<br>ELEQNIKELNTLADQVDTTHELLTKTSLVASSSGAVSGVMNILGLALAPVTAGGSLML<br>SATGTGLGAAAAITNIVTNVLENRSNSAARDKASRLGPLTTSHEAFGGINWSEIEAAG<br>FCVNKCVKAIQGIKDLHAYQMAKSNSGFMAMVKNFVAKRHIPFWTARGVQRAFEGT<br>TLAMTNGAWVMGAAGAGFLLMKDMSSFLQSWKHLEDGARTETAEELRALAKKLEQ<br>ELDRLTQHHRHLPQKASQTCSSSRGRAVRGSRVVKPEGSRSPLPWPVVEHQPRLG<br>PGVALRTPKRTVSAPRMLGHQPAPPAPARKGRQAPGRHRQ
**APOL6 (NP_085144.1)**<br>MDNQAERESEAGVGLQRDEDDAPLCEDVELQDGDLSPEEKIFLREFPRLKEDLKGNI<br>DKLRALADDIDKTHKKFTKANMVATSTAVISGVMSLLGLALAPATGGGSLLLSTAGQG<br>LATAAGVTSIVSGTLERSKNKEAQARAEDILPTYDQEDREDEEEKADYVTAAGKIIYN<br>LRNTLKYAKKNVRAFWKLRANPRLANATKRLLTTGQVSSRSRVQVQKAFAGTTLAM<br>TKNARVLGGVMSAFSLGYDLATLSKEWKHLKEGARTKFAEELRAKALELERKLTELT<br>QLYKSLQQKVRSRARGVGKDLTGTCETEAYWKELREHVWMWLWLCVCLCVCVYV<br>QFT

Supplementary Table 2.

Name	Primer sequence
APOL2 F<br>APOL2R	5' ATTCTAGAGCCACCATGAACCCAGAGAGCAGTATC 3'<br>5' ACTGGAATTCTCACTATTGGTCTTGGCCTGGCTGCAG 3'<br>Inserted into pRG977
APOL3F<br>APOL3R	5' AATCTAGAGCCACCATGGACTCAGAAAAGAAACGCTTTAC 3'<br>5' GATCGAATTCTCATCAGTGGGTATGGCATGGATTCAGAC 3'<br>Inserted into pRG977
APOL4F<br>APOL4R	5' AATCTAGAGCCACCATGGAGGGAGCTGC 3'<br>5' GATCGAATTCTCATTAGCCTGCTTTTAGACTCTGATG 3'<br>APOL4 v. without signal peptide<br>Inserted into pRG977
APOL5F<br>APOL5R	5' AATCTAGAGCCACCATGCCATGTGGCAAAC 3'<br>5' GATCGAATTCTCATTATTGTCGGTGTCTTCCCGGG 3'<br>Inserted into pRG977
APOL6F<br>APOL6R	5' AATCTAGAGCCACCATGGACAACCAGGC 3'<br>5' GATCGAATTCTCATCATGTAAACTGTACATACACACAGAC 3'<br>Inserted into pRG977
G-block primer for Myc-tag pRG977 at N-terminus	5'GTCTCATCATTTTGGCAAAGAATTTATGCCTCGAGGCTAGCGCCACCATGGAACAAAAACTCATCTCAGAAGAGGATCTGGGTGGCAGCGGAGGTGGAGGCTCAGGTGGCTCTAGAATGCTTTGGGAACCCGGGTCTCT 3'

G-block primer for APOL1 with signal peptide followed by Myc-tag at N-terminus	5’tatgcctcgaggctagcgccaccatgGAGGGAGCTGCTTTGCTGAGAGTCTCTGTCCTCTGCATCTGGATGAGTGCACTTTTCCTTGGTGTGGGAGTGAGGGCAatgcatgaacaaaaactcatctcagaagaggatctgggtggcagcggaggtggaggctcaggtggctctagaatgct 3’<br>Uppercase denotes APOL1 sequence
APOL1MycF<br>APOL1MycR	5’ATGCTCTAGAGAGGGAGCTGCTTTGCTGAG<br>5’ TCAACGAATTCTCACAGTTCTTGGTCCGCC 3’<br>APOL1 (O14791-1) inserted into tagged pRG977
APOL1 v.3 MycF<br>APOL1v.3 MycR	5’ ATGCTCTAGAGAGGAAGCTGGAGCGAGG 3’<br>5’ TCAACGAATTCTCACAGTTCTTGGTCCGCC 3’<br>(downstream of signal peptide cleavage site) APOL1 v.3 inserted into tagged pRG977.
APOL2MycF<br>APOL2MycR	5’ ATGCTCTAGAAACCCAGAGAGCAGTATCTTTATTG 3’<br>5’ ACTGGAATTCTCACTATTGGTCTTGGCCTGGCTGCAG 3’<br>APOL2 inserted into tagged pRG977
APOL3MycF<br>APOL3MycR	5’ ATGCTCTAGAGACTCAGAAAAGAAACGCTTTACTG 3’<br>5’ GATCGAATTCTCATCAGTGGGTATGGCATGGATTCAGAC 3’<br>APOL3 inserted into tagged pRG977
APOL4 MycF<br>APOL4MycR	5’ ATGCTCTAGAGAGGGAGCTGCTTTGCTGAAAATC 3’<br>5’ GATCGAATTCTCATTAGCCTGCTTTTAGACTCTGATG 3’<br>APOL4 v.2 inserted into tagged pRG977

APOL4NoSP MycF<br>APOL4NoSP MycR	5'ATGCTCTAGAGGATCCTGGGTGCAGCTCATC 3'<br>5' GATCGAATTCTCATTAGCCTGCTTTTAGACTCTGATG 3'<br>APOL4 v.1 inserted into tagged pRG977
APOL5MycF<br>APOL5MycR	5'ATGCTCTAGACCATGTGGCAAACAAGGAAATTTGC 3'<br>5' GATCGAATTCTCATTATTGTCGGTGTCTTCCCGGG 3'<br>APOL5 inserted into tagged pRG977
APOL6MycF<br>APOL6MycR	5'ATGCTCTAGAGACAACCAGGCGGAGAGAGAAAG 3'<br>5' GATCGAATTCTCATCATGTAAACTGTACATACACACAGAC 3'<br>APOL6 inserted into tagged pRG977

REFERENCES

1. Hofmann, K., and Stoffel, W. (1993) TMbase-A database of membrane spanning proteins segments. *Biol. Chem. Hoppe-Seyler* **374**, 166
2. Käll, L., Krogh, A., and Sonnhammer, E. L. (2004) A combined transmembrane topology and signal peptide prediction method. *J Mol Biol* **338**, 1027-1036
3. Käll, L., Krogh, A., and Sonnhammer, E. L. (2007) Advantages of combined transmembrane topology and signal peptide prediction--the Phobius web server. *Nucleic Acids Res* **35**, W429-432
4. Kyte, J., and Doolittle, R. F. (1982) A simple method for displaying the hydropathic character of a protein. *J Mol Biol* **157**, 105-132
5. Almagro Armenteros, J. J., Tsirigos, K. D., Sønderby, C. K., Petersen, T. N., Winther, O., Brunak, S., von Heijne, G., and Nielsen, H. (2019) SignalP 5.0 improves signal peptide predictions using deep neural networks. *Nat Biotechnol* **37**, 420-423
